# An updated re-analysis of the mortality risk from nasopharyngeal cancer in the National Cancer Institute formaldehyde worker cohort study

**DOI:** 10.1186/s12995-016-0097-6

**Published:** 2016-03-02

**Authors:** Gary M. Marsh, Peter Morfeld, Sarah D. Zimmerman, Yimeng Liu, Lauren C. Balmert

**Affiliations:** Center for Occupational Biostatistics and Epidemiology and Department of Biostatistics, Graduate School of Public Health, University of Pittsburgh, 130 DeSoto Street, Pittsburgh, PA 15261 USA; Institute and Policlinic for Occupational Medicine, Environmental Medicine and Preventive Research, University of Cologne, Cologne, Germany; Institute for Occupational Epidemiology and Risk Assessment of Evonik Industries, Essen, Germany

**Keywords:** Formaldehyde, Nasopharyngeal cancer, Cohort mortality study, Occupational health, National Cancer Institute, Reanalyses

## Abstract

**Background:**

To determine whether the National Cancer Institute’s (NCI) suggestion of a persistent increased mortality risk for nasopharyngeal cancer (NPC) in relation to formaldehyde (FA) exposure is robust with respect to alternative methods of data analysis.

**Methods:**

NCI provided the cohort data updated through 2004. We computed U.S. and local county rate-based standardized mortality ratios (SMRs) and internal cohort rate-based relative risks (RR) in relation to four formaldehyde exposure metrics (highest peak, average intensity, cumulative, and duration of exposure), using both NCI categories and alternative categorizations. We modeled the plant group-related interaction structure using continuous and categorical forms of each FA exposure metric and evaluated the impact of NCI’s decision to exclude non-exposed workers from the baseline category.

**Results:**

Overall, our results corroborate the findings of our earlier reanalyses of data from the 1994 NCI cohort update. Six of 11 NPC deaths observed in the NCI study occurred in Plant 1, two (including the only additional NPC death) occurred in Plant 3 among workers in the lowest exposure category of highest peak, average intensity and cumulative FA exposure and in the second exposure category of duration of exposure, and the remaining cases occurred individually in three of eight remaining plants. A large, statistically significant, local rate-based NPC SMR of 7.34 (95 % CI = 2.69–15.97) among FA-exposed workers in Plant 1 contrasted with an 18 % deficit in NPC deaths (SMR = 0.82, 95 % CI = .17–2.41) among exposed workers in Plants 2–10. Overall, the new NCI findings led to: (1) reduced SMRs and RRs in the remaining nine study plants in unaffected exposure categories, (2) attenuated exposure-response relations for FA and NPC for all the FA metrics considered and (3) strengthened and expanded evidence that the earlier NCI internal analyses were non-robust and mis-specified as they did not account for a statistically significant interaction structure between plant group (Plant 1 vs. Plants 2–10) and FA exposure.

**Conclusions:**

Our updated reanalysis provided little or no evidence to support NCI’s suggestion of a persistent association between FA exposure and mortality from NPC. NCI’s suggestion continues to be driven heavily by anomalous findings in one study plant (Plant 1).

**Electronic supplementary material:**

The online version of this article (doi:10.1186/s12995-016-0097-6) contains supplementary material, which is available to authorized users.

## Background

Formaldehyde (FA) is an important industrial chemical. Production in the U.S. and the European Union exceeds 10 million tons per year [1]. Adhesives and binders are produced from resins based on FA (e.g., for the manufacture of particle board, paper, and vitreous synthetic fibers), to make plastics and coatings, and FA is used in textile finishing [2]. FA is an intermediate in the production of many chemicals, and as formalin it is used as a disinfectant and preservative. In addition, FA is produced in combustion, e.g. in vehicle exhausts and tobacco smoke [2]. Also, FA is formed endogenously in humans [3].

In 2004, the International Agency for Research on Cancer (IARC) reclassified FA from a probable (Group 2A) [4] to a known human carcinogen (Group 1) [1] citing results for nasopharyngeal cancer (NPC) mortality from the follow-up through 1994 of the National Cancer Institute (NCI) formaldehyde cohort study [5]. Based on the same NCI findings, the Group 1 classification was upheld by IARC following the working group meeting for IARC Monograph Volume 100F [2]. Subsequently, the U.S. National Institute of Environmental Health Sciences National Toxicology Program changed the classification of formaldehyde from “anticipated to be carcinogenic in humans” to “known to be a human carcinogen” [6].

In contrast, in 2012, the Committee for Risk Assessment^1^ of the European Chemicals Agency^2^ disagreed with the proposal to classify FA as a known human carcinogen (Carc. 1A), proposing a lower but still protective category, namely as a substance which is presumed to have carcinogenic potential for humans (Carc. 1B)^3^. Thus, U.S. and European regulatory agencies currently disagree about the potential human carcinogenicity of FA. An overview of open issues and scientific discussions about the health effects of FA exposures is given in Bolt and Morfeld [7].

### The National Cancer Institute formaldehyde cohort study

In June 2013, the NCI published the findings of its update through 2004 of mortality from solid tumors among workers in the US industry-wide FA study [8]. This study includes 10 plants and represents the largest cohort study of workers with potential exposure to FA [9]. The purpose of the Beane Freeman et al. update was to extend the mortality follow-up through 2004 and to examine the associations among different exposure characterizations and mortality from several solid tumors. This study also included corrections by Beane Freeman et al. [10] to the earlier update of mortality through 1994 published in 2004 [5]. For an evaluation of the errors that lead to these corrections see Issues 1 and 2 in Marsh et al. [11]. Beane Freeman et al. [8] claim that a persistent increased risk remains for NPC mortality within the updated cohort associated with peak, average intensity and cumulative FA exposure metrics as reported in Hauptmann et al. [5], although this NPC risk was not reported by Blair et al. [9] in the original FA cohort analysis based on follow-up through 1979. The main conclusion from Beane Freeman et al. [8] is that the update through 2004 suggests a link between FA exposure and NPC mortality that is consistent with some case–control studies [12–17]. Aside from not statistically significantly elevated rate ratios for salivary gland cancer mortality, the authors observed no associations with mortality from other cancer types reported in other studies, including lung, laryngeal, nasal sinus and brain [2, 4].

In 2013, two of us (GM, PM) published a commentary [11] describing why we believe NCI’s interpretation regarding the persistent NPC risk is not consistent with available epidemiological evidence including: (1) data from the most recent update of the NCI cohort study [8]; (2) other large and recently updated cohort studies of FA-exposed workers [18–21]; (3) alternative analyses of the 1994 update of the NCI cohort study [22–24] or (4) the independent study of one of the NCI’s study plants (Plant 1) [25]. Plant 1, which historically has included the majority of the NPC deaths observed in the NCI cohort [5, 9], was also the focus of our reanalyses of the 1994 update of the NCI cohort [22, 23]. Plant 1, a plastics producing plant operating since 1943 in Wallingford, CT, includes 4261 workers or 17 % of the total NCI cohort of 25,619 workers. Regarding potential for FA exposure, Table 1 shows that workers in the Plant 1 cohort had a median average intensity of exposure (AIE) of 1.023 ppm compared to a range of median AIEs of 0.08 to 2.799 ppm for Plants 2–10.

**Table 1:** Selected characteristics and findings for 10 plants in 2004 update of NCI formaldehyde cohort study

UPitt (NCI) plant no.	1 (1)	2 (2)	3 (3)	4 (5)	5 (6)	6 (7)	7 (8)	8 (10)	9 (11)	10 (12)
Entry year	1943	1945	1949	1958	1957	1951	1938	1934	1956	1941
No. Subjects	4261	784	2375	1692	744	5248	4228	1679	1933	2675
Formaldehyde exposure										
% Subjects ever exposed	87.7	99.9	92.7	93.3	64.4	91	81.6	99.3	88.2	95
% Subjects ever in highest peak category	46.1	91.6	0	72.9	20.4	2	.4	1.1	9.3	69.7
Median AIE (ppm) ^a^	1.023	2.799	.112	.234	.196	.233	.080	.382	.400	.543
(5–95 %-tile)	.310–1.417	.300–3.927	.010–.222	.100–.596	.029–1.132	.033–.868	.020–.250	.100–2.000	.100–1.615	.216–1.124
Median Cum (ppm-years) ^a^	.9	19.0	.1	2.2	1.9	.7	.1	.6	.3	1.3
(5–95 %-tile)	.1–17.2	.4–86.5	.01–2.1	.06–11.9	.08–27.5	.01–16.3	.01–3.5	.03–12.0	.03–5.9	.05–16.4
Median Dur (years) ^a^	1.0	11.3	1.1	9.7	16.7	3.6	1.0	1.0	.8	2.3
(5–95 %-tile)	.1–24.4	.3–30.7	.1–20.3	.4–29.5	1.0–34.4	.1–31.3	.1–28.0	.1–25.0	.09–16.5	.1–29.2
Observed and expected deaths and SMRs for NPC										
Obs	6	1	2	0	0	0	1	0	0	1
SMR-US (Exp)	5.44** (1.1)	4.32 (.2)	3.01 (.7)	- (.4)	- (.2)	- (1.1)	.93 (1.1)	- (.4)	- (.3)	1.21 (.8)
(95 % CI)	2–11.85	.11–24.08	.36–10.87	0–9.03	0–18.63	0–3.42	.02–5.18	0–9.39	0–12.79	.03–6.72
SMR-local (Exp)	5.57** (1.1)	4.03 (.2)	7.60 (.3)	- (.5)	- (.2)	- (1.3)	1.24 (.8)	- (0)	- (.4)	1.01 (1.0)
(95 % CI)	2.04–12.12	.10–22.48	.92–27.46	0–7.30	0–21.09	0–2.82	.03–6.89	0–90.04	0–10.14	.03–5.63

^a^Based on exposed jobs only with no lag

** *p* < .01

Siew et al. [26] analyzed a study cohort of all 1.2 million economically active Finnish men born between 1906 and 1945 who participated in the national population census on December 31, 1970. The Finnish job-exposure matrix (FINJEM) was used to calculate occupational FA exposure estimates [27]. The authors analyzed 149 NPC cases and found no association with FA exposure. Although the exposure assessment is limited in this investigation, the large register based study by Sew et al. adds to the cohort studies that showed no elevated NPC risk after FA exposure.

Checkoway et al. [28] performed a re-analysis of the NCI cohort and evaluated associations between cumulative and peak formaldehyde exposure and lympho-hematopoietic malignancies, in particular myeloid leukemia. The authors did not address NPCs. We note that the US National Institute of Environmental Health Sciences National Toxicology Program judged in their decision on FA that “the evidence for nasopharyngeal cancer is somewhat stronger than that for myeloid leukemia” [6]. Thus, it is of specific interest to examine whether the “stronger evidence” for NPC is robust and can be confirmed or refuted in a re-analysis of the updated NCI cohort study [8].

### Main methodological issues

Our recent commentary also described several methodological issues in the most recent update of the NCI study that formed the basis for our reanalysis of the updated NCI cohort data on mortality from NPC [11]. In this paper, we addressed three methodological issues: (Issue 1) inappropriateness of excluding unexposed workers from the evaluation of exposure-response relationships; (Issue 2) the trend tests used in the NCI 2004 updates produce misleading results and may be mis-specified and (Issue 3) failure to recognize the important interaction structure between plant group (i.e., Plant 1 vs. Plants 2–10) and FA exposure reported by Marsh et al. [23]. We report here our updated reanalysis of the relationship between FA exposure and mortality from NPC using data from the 2004 update of the NCI FA cohort study.

## Methods

### Data preparation

We obtained a copy of the updated 2004 NCI formaldehyde cohort study data from NCI. The 2004 NCI cohort file contained the same demographic, work history and formaldehyde exposure data for 25,619 workers first employed at one of 10 industrial plants before January 1, 1966 as the file associated with NCI’s 1994 update (1994 NCI cohort file). We were informed by NCI that the only differences between the 1994 and 2004 NCI cohort files were the updated vital status, cause of death, and date of death variables. All event dates (e.g., birth, hire, termination, and death) were limited to month and year to protect subject confidentiality. Further details about the NCI study are provided in Beane Freeman et al. [8] and Blair et al. [9].

Due to the complexity of reformatting the earlier 1994 NCI cohort data file in 2005 to enable analysis with the OCMAP-Plus cohort analysis program [29], and the lack of a common ID (for confidentiality purposes), we matched all deceased employees from the 2004 NCI cohort data file to the 1994 OCMAP cohort data file on all possible variables. We matched 13,883 of 13,951 deaths, or 99.5 %, exactly to the 1994 OCMAP file. For the remaining 68 deaths, we manually selected the closest matches within the 1994 OCMAP file. After the matching was completed, we updated vital status, cause of death, and date of death information for all 13,951 deaths so that the mortality follow-up period was through 2004.

Additionally, we created a new OCMAP file from the 2004 NCI cohort file to ensure that our matched OCMAP file was accurate. This new OCMAP file contained only a portion of the variables as the reformatting was too complex and redundant. We subsequently performed extensive cross-checks and replicated key NCI findings to establish the comparability of the files. Our total person-year count differed by only 11.0 or 0.00001 % of the total person-years reported by NCI [8]. We also matched the plant-specific numbers of subjects, total deaths and deaths from NPC. Compared with Beane Freeman et al. [30], which provided more detailed information, we also matched exactly on median duration of follow-up years (42 years) and median length of employment (2.6 years).

Our general NPC analyses were based on the total of 11 NPC deaths reported in the NCI study. As in our original reanalyses [22, 23], and unlike Hauptmann et al. [5] and Beane Freeman et al. [8], we did not omit from our exposure–response analyses the one NPC death in Plant 11 that had been recoded to oropharyngeal cancer based on findings of a medical record confirmation reported by Lucas [31]. Our concerns about this partial correction of death certificate information are reported elsewhere [32].

### Statistical analyses

#### General methods

In general, our external and internal comparisons of NPC mortality in the NCI 2004 FA cohort were conducted along the lines of our previous reanalysis of NPC [22] and leukemia [33] in the NCI 1994 FA cohort. A main goal was to determine whether our earlier findings were corroborated in this updated reanalysis. Specific goals were to address the three methodological issues noted above, as described below Our results for NCI-replicated analyses differ slightly from those reported by Beane Freeman et al. [8] because the 2004 NCI cohort file did not include the day of events (dates of birth and death and work history dates). We estimated day of event by using the midpoint of the month (15). Also, differences for NCI-replicated exposure-response models occurred because we fit our models with exact conditional logistic regression; whereas, NCI used asymptotic Poisson regression models. Finally, as noted above, our exposure-response models used all 11 NPC deaths; whereas, NCI’s were based on 10 deaths. To facilitate comparison, we also present results based on these 10 NPC deaths only.

#### External mortality comparisons for NPC

For NPC, we computed both U.S. and regional (local county) rate-based SMRs and their 95 % confidence intervals (CI, based on the Poisson distribution) by each of the 10 plants in the NCI study and by two plant groups (Plant 1 vs. Plants 2–10). SMRs were standardized for race/ethnicity, sex, age group, and time period. Local county area mortality rates for each of the 10 plants in the NCI study were obtained from the Mortality and Population Database System (MPDS) developed at the University of Pittsburgh [34]. For each study plant, the local county area was defined as the county or group of counties surrounding the plant from which most of the work force was drawn (see Marsh and Youk [34] for plant code, plant locations and counties comprising the regional rates). Because MPDS rates are not available before 1950, we applied 1950–1954 rates to previous observation periods for plants that started before 1950. This approximation should have negligible effect on SMRs, as only 3.3 % of the total person-years at risk in the cohort occurred before 1950 [34]. The proportional contribution of expected NPC deaths is likely to be even smaller because these early person-years are associated with relatively young age groups.

We also computed regional rate-based SMRs and 95 % CIs for NPC by each of the four formaldehyde metrics (highest peak, average intensity, cumulative, and duration) used in the NCI study^4^. We used the NCI exposure categories for highest peak exposure (the NCI data were pre-coded into fixed categories) and an alternative categorization for the remaining metrics (approximate tertiles of formaldehyde exposure among all NPC deaths in exposed workers, UPitt categories). Unlike the approximate 60th and 80th percentile cutpoints used by NCI, our categorization produces a more even distribution of NPC deaths among the exposed categories. When evaluating NCI exposure categories we used only 10 NPC deaths as the NCI researchers did in their analyses. We used all 11 NPC deaths in analyses applying UPitt categories.

#### Internal mortality comparisons

In the NCI study, Poisson regression based on asymptotic estimation was used to examine exposure–response relationships by comparing internal cohort rates for NPC. Alternatively, we used relative risk (RR) regression modeling with both exact and asymptotic estimation to investigate the dependence of the internal cohort rates (modeled as time to death) for NPC on combinations of both categorical and continuous formaldehyde metrics, with adjustment for potential confounding factors through matching or stratification. Study data from the entire 1934–2004 period were modeled. Risk sets were explicitly constructed from the cohort data with age as the primary time dimension, using the RISKSET program module in OCMAP-Plus [29]. To adjust for year of birth (“cohort” or time period) effects, risk sets were caliper-matched, within one year, on date of birth. Regression models included terms for race/ethnicity (white/black), sex, and payroll category (wage, salary, unknown) to adjust for these potential confounding factors. Trends in RRs relative to the exposure measures considered were based on likelihood ratio tests using either exposed workers or unexposed and exposed workers.

Relative risk regression models were fit using exact and asymptotic conditional logistic regression. The conditional logistic regression likelihood is equivalent to the partial likelihood of Cox regression [35] which can be understood as a refinement of Poisson regression [36]. While the exact models are more appropriate for the small numbers of NPC deaths involved in this analysis, we also ran asymptotic models to enable more direct comparisons with the asymptotic Poisson regression models run by Beane Freeman et al. [8]. Categorical FA exposure models were run in Stata/SE 13.1 [37] and continuous FA exposure models were run in SAS 9.4 [38]. The internal comparisons used the same exposure metric categorization scheme described for the external comparisons. All formaldehyde exposure metrics in the external and internal mortality comparisons incorporated the same 15-year lag period used by NCI. We addressed Methodological Issues 1, 2, and 3 within the internal comparisons.

##### Methodological issue 1

For Issue 1, we conducted and compared exposure-response analyses using both the lowest FA exposure category (as done by NCI) and the unexposed exposure category as the baseline. We argued (Issue 4 in [23]) that it is inappropriate to exclude unexposed workers from internal analyses as done by Beane Freeman et al. [30]. All workers are from the same factories and, as noted by McLaughlin et al. [39] in a response to a letter by Hauptmann and Ronckers [40], lagging of FA exposure by 15 years results in contributions to the unexposed category from workers who were, in fact, exposed to FA. Indeed, most of the person-time at risk allocated to the unexposed category represents years of follow-up of workers who were eventually exposed to FA. Therefore, it is of major interest to examine whether the statistically significant positive associations between FA exposure and NPC deaths as described by NCI in Bean Freeman et al. [8] can be replicated if unexposed workers are not dropped prior to analyses.

##### Methodological issue 2

For Issue 2, we avoided the problems associated with using continuous variable trend tests for categorical variables (as done by Beane Freeman et al. [8]; see Issue 5 in Marsh et al. [11] for background and details), by properly matching the trend test with the method of analysis. That is, we modeled the continuous form of the FA metrics (excluding highest peak) to produce a slope estimate that was evaluated for statistical significance (linear trend) via a likelihood ratio test. We used the actual continuous FA exposure in our asymptotic models (referred to in tables as Score 4); whereas, due to the computationally intensive permutation methods inherent in our exact models, we used the median FA exposure value associated with each category of the corresponding pseudo-continuous FA exposure metric (Score 3). As in our earlier reanalysis [23], a pseudo-continuous form of the NCI highest peak exposure variable was defined by scoring each of the categories used by Beane Freeman et al. [8] with the arithmetic mean of the interval, including a reasonable assumption about the score for the last open-ended interval (Scores: unexposed = 0, >0–1.9 = 0.95, 2.0–3.9 = 3.0, 4.0 + = 6.0) (Score 2). Likewise, we also modeled the categorical form of each FA metric and evaluated trend via a likelihood ratio test based on the category-specific score statistics. For all exposure metrics, we used the scores 1, 2, 3, 4 to represent the four categories of FA exposure, respectively (Score 1). In each exposure-response analysis, the main effect of the corresponding exposure metric was assessed with a global test.

##### Methodological issue 3

The key finding in our previous reanalyses of NPC mortality in the 1994 NCI cohort [22] was that NCI’s earlier conclusion of a possible causal association between FA exposure and NPC mortality risk [5] was driven heavily by a large, statistically significant excess in NPC mortality risk for employees from Plant 1. In a later reanalysis using a continuous form of the highest peak FA exposure metric, Marsh et al. [23] showed that the internal analyses of Hauptmann et al. [5] were non-robust and mis-specified as the authors did not account for a statistically significant interaction structure between plant group (Plant 1 vs. Plants 2–10) and highest peak FA exposure. Subsequently, to address plant heterogeneity for NPC mortality risks in the 2004 NCI FA cohort, Beane Freeman et al. [8] refuted the findings of Marsh et al. [23], and through an “influence analysis” concluded that they found “. . .*no evidence of plant heterogeneity for a broad group of metrics, including peak exposure.”* We maintain that Beane Freeman et al. [8] neither correctly interpreted the results of their own influence analysis nor correctly interpreted the results of the interaction evaluation performed by Marsh et al. [23]. For more details see Issue 6 in Marsh et al. [11].

To further address this issue of interaction structure using the 2004 NCI FA cohort data, we extended our earlier models [23], which were based only on the continuous form of the highest peak FA exposure metric, to include continuous forms of the other FA metrics considered (duration of exposure, average intensity of exposure and cumulative exposure). We also considered both exact and asymptotic estimation as described above. Our continuous form of the highest peak FA exposure metric described above enabled the fitting of an interaction term with the plant group indicator despite analyzing sparse data. We fit different specifications of the interaction model and modeled the continuous form peak exposure metric for Plant 1 and Plants 2–10 separately to gain insights into the meaning of the interaction terms derived from models based on all plants. Finally, we fit interaction models based on the plant group indicator and average FA exposure intensity, cumulative FA exposure or duration of FA exposure. We always evaluated all 11 NPC deaths in these models addressing the interaction issue.

## Results

### Statistical analyses - external mortality comparisons

Table 1 shows for each of the 10 NCI study plants, selected demographic and FA exposure characteristics and findings from the external mortality comparisons. Because the NCI 2004 update did not include new subjects nor extended work history information, the FA exposure characteristics are identical to those we discussed in our previous reanalysis [22]. We refer to plants by the sequential (UPitt) plant only.

Table 1 shows that the one additional NPC death observed in the 2004 NCI update occurred in Plant 3 resulting in not statistically significant U.S. and local-rate based SMRs of 3.01 and 7.60, respectively, based on two observed deaths. Because of this, U.S. and local rate-based SMRs for NPC in the remaining nine plants decreased from the 1994 update [22]. In particular, six of now 11 NPC deaths occurred in Plant 1 yielding statistically significant (*p* < .01) 5.44-fold and 5.57-fold excesses based on the U.S. and regional comparisons, respectively. In the 1994 update, the corresponding NPC SMRs were 6.62 (*p* < .01) and 7.39 (*p* < .01). The remaining three NPC deaths were scattered individually across three plants (Plants 2, 7, and 10), yielding not statistically significant local rate-based mortality excesses ranging from 1.01-fold (Plant 10) to 4.03–fold (Plant 2). No NPC deaths were observed in Plants 4–6 or 8–9.

Table 2 presents similar data as Table 1 for two plant groups (Plant 1 and Plants 2–10). The now five NPC deaths combined in Plants 2–10 yield a null finding (SMR = 1.06) compared with a statistically significant 5.57-fold excess for Plant 1 based on local NPC rates. An even greater difference in NPC local rate-based SMRs was observed between formaldehyde-exposed workers in Plant 1 (SMR = 7.34, 95 % CI = 2.69–15.97) and Plants 2–10 (SMR = 0.82, 95 % CI = 0.17–2.41), and the NPC SMR among unexposed workers in Plants 2–10 (SMR = 1.88, 95 % CI = 0.23–6.80) was more than twice that among the exposed workers (SMR = 0.82, 95 % CI = 0.17–2.41).

**Table 2:** Characteristics and findings for Plant 1 (Wallingford) and Plants 2-10 combined in 2004 NCI update

Characteristic/finding	Plant 1 (Wallingford)	Plant 2–10 (all other plants)
Entry year	1940	1934–1958
No. subjects	4261	21358
Formaldehyde exposure		
% Subjects ever exposed	87.7	89.8
% Subjects ever in highest peak category	46.1	20.1
Median AIE (ppm) ^a^	1.023	0.366
(5–95 %-tile)	(.310–1.417)	(.052–1.257)
Median Cum (ppm-years) ^a^	.9	3.2
(5–95 %-tile)	(.1–17.2)	(.06–23.5)
Median Dur (years) ^a^	1.0	13.1
(5–95 %-tile)	(.1–24.4)	(.3–32.1)
Observed deaths and SMRs		
*All workers*		
Observed deaths	6	5
SMR-US (expected deaths)	5.44** (1.1)	.97 (5.2)
(95 % CI)	(2–11.85)	(.31–2.26)
SMR-local (expected deaths)	5.57** (1.1)	1.06 (4.7)
(95 % CI)	(2.04–12.12)	(.35–2.48)
*Exposed workers*		
Observed deaths	6	3
SMR-US (expected deaths)	7.23** (.8)	.74 (4.1)
(95 % CI)	(2.65–15.74)	(.15–2.16)
SMR-local (expected deaths)	7.34** (.8)	.82 (3.6)
(95 % CI)	(2.69–15.97)	(.17–2.41)
*Unexposed workers*		
Observed deaths	0	2
SMR-US (expected deaths)	- (.3)	1.81 (1.1)
(95 % CI)	(0–13.55)	(.22–6.53)
SMR-local (expected deaths)	- (.3)	1.88 (1.1)
(95 % CI)	(0–14.20)	(.23–6.80)

^a^Based on exposed jobs only with no lag

** *p* < .01

Table 3 shows local rate-based NPC SMRs for each of the four FA exposure metrics for all plants combined and by two plant groups (Plant 1 and Plants 2–10). To facilitate comparison, results from the 1994 [22] and 2004 NCI updates are shown side-by-side. In addition to the reasons noted in the Methods section, SMRs differ between the corresponding NCI and UPitt analyses due to the alternative UPitt categorizations used for all but highest peak exposure. Table 3 shows that the one additional NPC death observed in Plant 3 (Table 1) occurred in the lowest FA exposure category (Exp Cat 1) of each of the metrics considered except for duration of exposure (Exp Cat 2). For all plants combined and Plants 2–10, this finding led to an increased SMR for NPC in the corresponding categories. In Plant 1 and for all other categories that did not include the additional death, NPC SMRs decreased between the 1994 and 2004 updates. Similar to our earlier findings [22], SMRs in Table 3 for all plants combined are elevated for nearly all unexposed and exposed categories of each metric considered and are statistically significant for the highest exposure categories of highest peak exposure, average intensity of exposure, and cumulative exposure (UPitt categories only). Many SMRs in the baseline (unexposed) categories exceed those in the corresponding non-baseline categories.

**Table 3:** NCI FA cohort, NPC SMR results, local comparisons, by FA exposure, update and plant group

	Highest Peak Category^a^						AIE^b^							Cumulative Exposure (Cum)^b^						Duration of Exposure (Dur)^b^					
Metric^c,d^	2004 NCI			1994 NCI ^f^			2004 NCI			1994 NCI ^f^			Metric^c,d^	2004 NCI			1994 NCI ^f^			2004 NCI			1994 NCI ^f^		
	Obs	SMR^e^	95 % CI	Obs	SMR^e^	95 % CI	Obs	SMR^e^	95 % CI	Obs	SMR^e^	95 % CI		Obs	SMR^e^	95 % CI	Obs	SMR^e^	95 % CI	Obs	SMR^e^	95 % CI	Obs	SMR^e^	95 % CI
All Plants													All Plants												
NCI Cats.													NCI Cats.												
Unexposed	2	1.98	(0.24–7.16)	2	2.22	(0.27–8.00)	2	1.51	(0.18, 5.46)	2	1.62	(0.20–5.84)	Unexposed	2	1.51	(0.18–5.46)	2	1.62	(0.20–5.84)	2	1.51	(0.18–5.46)	2	1.62	(0.20–5.84)
Exp Cat 1	1	1.03	(0.03–5.73)	0	0	(0.00–2.46)	1	0.41	(0.01, 2.28)	0	--	(0.00–1.77)	Exp Cat 1	4	1.50	(0.41–3.83)	3	1.36	(0.28–3.97)	5	1.83	(0.59–4.26)	4	1.80	(0.49–4.62)
Exp Cat 2	0	--	(0.00, 2.24)	0	0	(0.00–3.47)	1	0.91	(0.02, 5.09)	1	1.17	(0.03–6.50)	Exp Cat 2	1	1.05	(0.03–5.82)	1	1.25	(0.03–6.98)	1	0.98	(0.02–5.45)	1	1.07	(0.03–5.96)
Exp Cat 3	7	3.89**	(1.56–8.01)	7	4.84**	(1.94–9.97)	6	6.67**	(2.81, 14.42)	6	8.36**	(3.07–18.21)	Exp Cat 3	3	3.75	(0.77–10.96)	3	4.57	(0.94–13.37)	2	2.86	(0.35–10.32)	2	3.94	(0.48–14.25)
UPitt Cats.													UPitt Cats.												
Unexposed	2	1.98	(0.24–7.16)	2	2.22	(0.27–8.00)	2	1.51	(0.18, 5.46)	2	1.62	(0.20–5.84)	Unexposed	2	1.51	(0.18–5.46)	2	1.62	(0.20–5.84)	2	1.51	(0.18–5.46)	2	1.62	(0.20–5.84)
Exp Cat 1	1	1.03	(0.03–5.73)	0	--	(0.00–2.46)	4	1.11	(0.30, 2.83)	3	0.99	(0.20–2.90)	Exp Cat 1	4	1.86	(0.51–4.77)	3	1.69	(0.35–4.94)	3	2.32	(0.48–6.77)	3	2.88	(0.40–8.43)
Exp Cat 2	0	--	(0.00, 2.24)	0	--	(0.00–3.47)	2	6.33	(0.77, 22.86)	2	7.60	(0.92–27.46)	Exp Cat 2	2	1.08	(0.13–3.89)	2	1.30	(0.16–4.68)	3	1.83	(0.38–5.36)	2	1.49	(0.18–5.38)
Exp Cat 3	8	4.50**	(1.94–8.87)	8	5.53**	(2.39–10.90)	3	5.73*	(1.18, 16.75)	3	8.06*	(1.66–23.55)	Exp Cat 3	3	6.60*	(1.36–19.30)	3	8.80*	(1.82–25.73)	3	1.96	(0.41–5.74)	3	2.35	(0.48–6.86)
Plant 1													Plant 1												
UPitt Cats.													UPitt Cats.												
Unexposed	0	--	(0.00, 2.09)	0	0	(0.00–24.59)	0	--	(0.00, 14.20)	0	--	(0.00–15.97)	Unexposed	0	--	(0.00–14.20)	0	--	(0.00–15.97)	0	--	(0.00–14.20)	0	--	(0.00–15.97)
Exp Cat 1	0	--	(0.00, 2.90)	0	--	--	2	4.88	(0.59, 17.61)	2	7.46	(0.90–26.94)	Exp Cat 1	3	8.26*	(1.70–24.14)	3	11.70**	(2.41–34.18)	3	9.14**	(1.89–26.72)	3	12.79**	(2.64–37.37)
Exp Cat 2	0	--	(0.00, 2.90)	0	--	(0.00–13.54)	2	10.74*	(1.30, 38.79)	2	13.96*	(1.69–50.44)	Exp Cat 2	2	5.24	(0.63–18.93)	2	7.21	(0.87–26.04)	2	6.30	(0.76–22.75)	2	9.01*	(1.09–32.54)
Exp Cat 3	6	12.91**	(4.74–28.10)	6	17.04**	(6.25–37.08)	2	9.03*	(1.09, 32.64)	2	11.78*	(1.43–42.57)	Exp Cat 3	1	13.66	(0.34–76.10)	1	21.18	(0.53–118.03)	1	5.80	(0.15–32.34)	1	8.03	(0.20–44.75)
Plants 2–10													Plants 2–10												
UPitt Cats.													UPitt Cats.												
Unexposed	2	2.42	(0.29–8.75)	2	2.66	(0.32–9.60)	2	1.88	(0.23, 6.80)	2	1.99	(0.24–7.18)	Unexposed	2	1.88	(0.23–6.80)	2	1.99	(0.24–7.19)	2	1.88	(0.23–6.80)	2	1.99	(0.24–7.18)
Exp Cat 1	1	1.04	(0.03–5.77)	0	--	(0.00–2.46)	2	0.62	(0.08, 2.25)	1	0.36	(0.01–2.02)	Exp Cat 1	1	0.56	(0.01–3.12)	0	--	(0.00–2.43)	0	--	(0.00–3.82)	0	--	(0.00–4.58)
Exp Cat 2	0	--	(0.00, 2.90)	0	--	(0.00–4.66)	0	--	(0.00, 28.43)	0	--	(0.00–30.78)	Exp Cat 2	0	--	(0.00–2.50)	0	--	(0.00–2.92)	1	0.76	(0.02–4.23)	0	--	(0.00–3.29)
Exp Cat 3	2	1.52	(0.18–5.50)	2	1.83	(0.22–6.60)	1	3.31	(0.08, 18.45)	1	4.94	(0.12–27.50)	Exp Cat 3	2	5.25	(0.64–18.96)	2	6.81	(0.82–24.61)	2	1.48	(0.18–5.33)	2	1.73	(0.21–6.26)

^a^NCI categories based on 60 and 80th percentiles of formaldehyde exposure among cancer deaths who were exposed. Includes only 10/11 deaths

^b^University of Pittsburgh categories based on approx. tertiles of formaldehyde exposure among NPC deaths who were exposed. Include 11 deaths

^c^All exposures lagged 15 years as in NCI study

^d^NCI exposure category cutpoints: highest peak (>0- < 2.0, 2.0- < 4.0, and 4.0+ ppm); average intensity of exposure (>0-0.5, 0.5- < 1.0, and 1.0+ ppm); cumulative exposure (>0-1.5, 1.5- < 5.5, and 5.5+ ppm-years); duration of exposure (>0- < 5.0, 5.0- < 15.0, and 15.0+ years). UPitt exposure category cutpoints: highest peak (same as NCI) (>0- < 2.0, 2.0- < 4.0, and 4.0+ ppm); average intensity of exposure (<1.046, 1.046-1.177, and 1.178+ ppm); cumulative exposure (<0.734, 0.734-10.150, and 10.151+ ppm-years); duration of exposure (<0.617, 0.617-6.263, and 6.264+ years)

^e^All SMRs adjusted for sex, race, age group, and time period

^f^From Marsh and Youk (2005)

*p < 0.05, **p < 0.01

The pattern of NPC SMRs for Plant 1 is similar to those reported in the independent study of Plant 1 [25, 41, 42], namely, very large and often statistically significant excesses in NPC across all non-baseline exposure categories, but little evidence of consistent exposure–response relationships across the formaldehyde exposure metrics considered. All NPC deaths in Plant 1 occurred among exposed workers. For highest peak exposure in Plant 1, all six NPC deaths occurred in the greatest exposure category (4 + ppm) yielding a lower but statistically significant (*p* < .01) SMR of 12.91 (95 % CI = 4.74–28.10). In contrast, for Plants 2–10 combined, two of the five (or two of four for NCI) NPC deaths occurred among workers unexposed to formaldehyde yielding a near 2-fold or greater NPC excess in each of the four baseline categories. For two metrics (highest peak and duration of exposure) the baseline NPC SMR exceeded that observed among the most highly exposed workers.

### Statistical analyses - internal mortality comparisons

Additional file 1: Table S1a-d show the results of our internal, exact relative risk (RR) regression analysis for NPC for each of the four FA metrics considered (highest peak, average intensity, cumulative and duration, respectively). Each table shows results for all plants combined, Plant 1 and Plants 2–10, and using both the unexposed category (left portion) and lowest exposure category (right portion) as the baseline category for RR estimates. Also shown are results for each sub-analysis using the NCI categories (based on 10 NPC deaths) [8] and our alternative FA exposure categorization (based on 11 NPC deaths). Each sub-analysis shows slope estimates and corresponding p-values for both categorical and continuous (or pseudo-continuous) forms of the FA metrics considered (Scores 1–4 as noted above), as well as the global test p-value.

Our concern about the inappropriateness of omitting unexposed workers from the baseline category in exposure-response analyses (Issue 1) is evident in the results presented in Additional file 1: Table S1a-d. This became especially problematic in the 2004 NCI update, as the only additional NPC death observed occurred in the lowest exposure category used by NCI as the baseline for comparison. Specifically, Additional file 1: Table S1a-d show for each of the four FA metrics considered and for all Plants combined and Plants 2–10, a marked difference in results using the lowest exposure category (Exp Cat 1) baseline compared to using the unexposed as baseline, which we believe is more appropriate. Using Exp Cat 1 as baseline, RRs for NPC in the unexposed category (compared with Exp Cat 1) were consistently elevated and for Plants 2–10 often exceeded the RR for the higher two exposure categories (Exp Cats 2–3).

Further, by omitting the unexposed from the assessment of exposure-response, there appears to be some evidence of a trend in RRs with increasing FA exposure based on Exp Cats 1–3 for highest peak (NCI categories: RRs = 4.05, 1.00 (baseline), 1.27, 7.23; Additional file 1: Table S1a) and AIE (NCI categories: RRs = 6.33, 1.00 (baseline), 2.54, 11.29; Additional file 1: Table S1b), as evident by the Scores 1–3 based trend tests^5^. These results lead NCI to conclude that the association between FA and NPC persisted in the 2004 update. We observed similar, yet less pronounced, differences based on our categorization of highest peak and AIE.

**Fig. 1:**
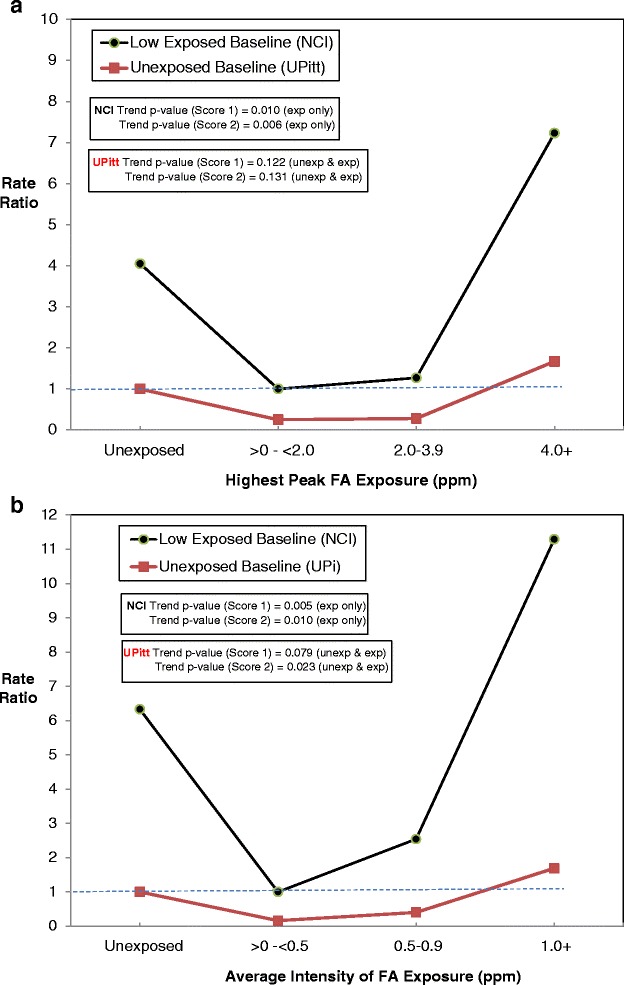
**a** RRs and 95 % CIs by Highest Peak Formaldehyde Exposure, Exact Estimation, (from Additional file 1: Table S1a). **b** RRs and 95 % CIs by Average Intensity of Formaldehyde Exposure, Exact Estimation, (from Additional file 1: Table S1b)

Conversely, for each of the four FA metrics, our corresponding analyses using unexposed as baseline yielded lower RRs for Exp Cats 1–3 and little or no evidence of an exposure-response association for highest peak (NCI categories: RRs = 1.00 (baseline), 0.25, 0.28, 1.67; Additional file 1: Table S1a), AIE (NCI categories: RRs = 1.00 (baseline), 0.16, 0.40, 1.69; Additional file 1: Table S1b) or the other FA metrics considered. While our Score 3 based trend test using the NCI categories for AIE was statistically significant (*p* = .023, Additional file 1: Table S1b), this was based on an unimportant U-shaped distribution of RRs for Exp Cat 1–3. Again, we observed similar, yet less pronounced, differences based on our categorization of highest peak and AIE. Figure 1a, b illustrate using NCI categories the influence of the baseline category on the results for highest peak and AIE, respectively.

Because the one additional NPC death in the NCI 2004 update occurred in Plant 3 (Table 1), the results of our reanalyses of Plant 1 (Additional file 1: Table S1a-d) did not change markedly from those presented earlier [22]. Our results reinforce, however, our earlier findings that the results for all plants combined discussed above, are heavily driven by Plant 1 where 5 of 10 NPC deaths (NCI analysis) or 6 of 11 (our main analysis) occurred [22].

The results of our exposure-response analyses based on asymptotic models (Additional file 2: Table S2a–d) were generally consistent with the corresponding exact models. Most trend test p-values decreased slightly compared to the exact analyses, yet the conclusions are consistent. Only the trend test of pseudo-continuous form of highest peak exposure (Score 2) were statistically significant for all analysis among both exposed and non-exposed workers (UPitt categories: *p* = .02, Additional file 2: Table S2a) and this was based on an unimportant U-shaped distribution of RRs for the unexposed and Exp Cat 1–3. Moreover, our results based on asymptotic conditional logistic regression are similar to those of Beane Freeman et al. [8], who used asymptotic Poisson regression. For example, in their paper, the overall RR estimate for the unexposed group using low exposure as baseline is 4.39 (95%CI: 0.36–54.05) for highest peak exposure, 6.79 (95%CI: 0.55–83.64) for average intensity of exposure and 1.87 (0.30–11.67) for cumulative exposure. These results are compared to our results in Additional file 2: Table S2 a-c with low exposed group as baseline (4.35 (95%CI: 0.35–54.40), 6.73 (95%CI: 0.53–85.19) and 1.86 (95%CI: 0.29–11.84), respectively.

### Statistical analyses - confounding and interaction of NPC risk estimates: the role of plant group

Additional file 3: Table S3a presents the findings of nine different internal modeling approaches of NPC risk in the NCI cohort, evaluating highest peak exposure to FA. All models were adjusted for the standard covariates age, time, sex, race, and payroll category. The regression model findings are described below in terms of the model numbers in the first column of Additional file 3: Table S3a. In Models 1 and 2, the number of observed deaths, the relative risk estimates, the estimated 95 %-confidence intervals (CI) of the relative risks and likelihood ratio trend p-values are shown. Model 2 was adjusted additionally for plant group (Plant 1 vs. Plants 2–10).

The results of Models 1–2 are similar, showing elevated RRs only in the highest exposure category, although far from being statistically significant. After adjustment for plant group, the RR estimate in the highest category was reduced (the estimated relative risk dropped from 1.8 to 1.4 after adjustment). As performed by NCI [8], trend tests (termed Score 1 trend tests here) were based on the full cohort as well as on exposed subjects only. Statistically significant Score 1 trends could only be found if the analysis was restricted to exposed workers. The Score 1 trend p-values were larger than 0.05 when analyzing the full cohort. In Models 3–9, the pseudo-continuous form of the highest peak exposure variable (Score 2) was evaluated in the exact analysis. We report coefficients (βs) of the regression models and the relative risks (RR) linked to coefficients via the formula, exp(β) = RR.

One possible way to identify ill-fitted models is to compare p-values calculated for the same models by different algorithms: the global likelihood ratio p-values shown in the last column of Models 3 to 9 are similar to the Wald p-values (presented together with the 95 %-CIs). Therefore, the model sequence fitted with continuous highest peak FA exposure did not reveal problems when using this p-value criterion.

An analysis similar to categorical Models 1 and 2 is presented in Models 3 and 7 analyzing continuous form highest peak FA exposure (pseudo-continuous highest peak Score 2). In Model 3, only the standard covariates (sex, race & payroll category) were used; Model 7 adjusted additionally for plant group. Again, the effect estimate was somewhat reduced after adjustment, although not remarkably (the coefficient decreased from 0.31 to 0.26 after adjustment, which corresponds to a relative risk reduction from 1.37 to 1.29). The plant group indicator showed a relationship with NPC risk after adjustment for continuous highest peak exposure (global *p* = 0.08, Model 7) and significantly so without adjustment for exposure (*p* = 0.015, Model 6), indicating a higher risk at Plant 1. After Model 6 was extended by an interaction term of continuous highest peak exposure and plant group indicator (shown in Model 9 of Additional file 3: Table S3a), the analysis became much more unstable. The plant group indicator variable and the interaction term were accompanied by confidence interval limits that spread out to infinity on one side each. However, the likelihood ratio test returned a p-value of 0.09 for the interaction term (the Wald test p-value was 0.08). A positive interaction between exposure and plant group is indicated. Analyzing the continuous form highest peak variable separately in both plant groups (Models 4 and 5 of Additional file 3: Table S3a) reproduced this finding from the interaction model: the formaldehyde highest peak exposure effect appears to be restricted to Plant 1 only (*p* = 0.05).

In contrast, for Plants 2–10 the likelihood ratio p-value was 0.67, clearly indicating that the effect was far from being statistically significant in these plants. Accordingly, the coefficient of exposure was estimated to be almost negligible in Plants 2–10 in comparison to Plant 1 (Model 4: 0.0072 vs. Model 5: 0.64). In addition, the confidence interval of the coefficient for Plants 2–10 was situated rather symmetrically around the null. After dropping the main effect of plant group from the full interaction model (full model = Model 9) the estimation process yielded more stable findings (reduced model = Model 8): the interaction between plant group indicator and continuous form highest peak FA exposure was now found to be significant (*p* = 0.03). When fitting asymptotic models (Additional file 4: Table S4a) we obtained similar results for the real continuous highest peak FA exposures with p-values being smaller than in the exact analyses. The global p-values for the interaction terms were 0.011 (Model 8) and 0.015 (Model 9).

Additional file 3: Table S3 b, c, d show results after repeating the exact analyses evaluating average intensity, cumulative exposure or duration of exposure to FA. For all three metrics, we used the continuous-form exposure Score 3 in Model 3–5 and Model 7–9. Model 8 indicate interactions between the plant group variable and the exposure metric in all three analyses: likelihood ratio p-values were 0.06 (average intensity, Additional file 3: Table S3b), 0.004 (cumulative exposure, Additional file 3: Table S3c), and 0.005 (duration of exposure, Additional file 3: Table S3d). We note that findings on cumulative exposure are more unstable because Wald p-values and likelihood ratio p-values differ considerably in many of the returned model findings.

The results of our asymptotic models (Additional file 4: Table S4a b, c, d) were generally consistent with those in the exact analysis. Similar to the asymptotic analysis of highest peak exposure, most of the p-values in the asymptotic analysis of the average intensity, cumulative exposure and duration of exposure to FA decreased compared to the exact analysis. Models 8 indicate interactions between the plant group variable and the corresponding exposure metric in all three asymptotic analyses: likelihood ratio p-values were 0.007 (average intensity, Additional file 4: Table S4-b), 0.015 (cumulative exposure, Additional file 4: Table S4-c), and 0.088 (duration of exposure, Additional file 4: Table S4-d).

## Discussion

In this paper, we challenged NCI’s claim that an increased mortality risk for nasopharyngeal cancer (NPC) in relation to formaldehyde (FA) exposure persisted in their 2004 update of the FA cohort [8]. As we demonstrated in our re-analyses of the 1994 update of the NCI FA cohort [22, 23], and again here, NCI’s claim of a persistent NPC risk stemmed from the use of inappropriate and non-robust statistical analysis methods. The foundation of our current reanalyses was three of the six methodological issues presented earlier: inappropriateness of excluding unexposed workers from exposure-response evaluations; improper trend tests and failure to recognize the important interaction structure between Plant 1 and Plants 2–10 [11].

Our reanalyses included external mortality comparisons via SMRs, in which we compared NPC rates among workers with the corresponding NPC rates of the general populations of both the U.S. and regional CT area. This enabled comparison with NCI’s U.S. rate-based only SMRs, and provided new data that accounted for geographic variability in NPC rates. Our reanalyses also included comparisons of NPC mortality among subgroups of workers defined by FA exposure level. In these exposure-response evaluations, we fit relative risk regression models in which subgroups of workers with higher FA exposure were compared to workers with lower or no FA exposure.

We fit many variations of our models to address the three issues noted above. For example, we used both the lowest FA exposure category (as done by NCI) and the unexposed category (our recommended approach) as the baseline category. We also modeled the continuous forms (i.e., not categorized) of the FA exposure metrics and applied corresponding continuous variable trend tests. This enabled a comparison with NCI, where continuous variable trend tests were inappropriately applied to categorical FA exposure variables. Further, to address the dramatic difference in NPC mortality among workers in Plant 1 vs. Plants 2–10, we fit models that included terms to account for this important interaction structure. To date, NCI has not fit models that account explicitly for this interaction. Finally, because NCI relied on Poisson regression based on asymptotic estimation rather than relative risk regression to evaluate exposure-response relationships for FA and NPC, we fit our models using both asymptotic and exact estimation, the latter being better suited for the small number of observed NPC deaths.

Overall, our reanalyses of the 2004 update of the NCI FA cohort do not support an association between FA exposure and NPC as suggested by Hauptmann et al. [5] and Beane Freeman et al. [8]. Our findings and conclusion also corroborate those presented in our earlier reanalysis of the NCI 1994 FA cohort data, and are now even stronger given that the one additional NPC death observed by NCI occurred in Plant 3 among workers in the lowest exposure category of highest peak, average intensity and cumulative FA exposure and in the second exposure category of duration of exposure. This finding led to: (1) reduced SMRs and RRs in the remaining nine study plants in unaffected exposure categories, (2) attenuated exposure-response relations for FA and NPC for all the FA metrics considered and (3) strengthened and expanded evidence that the internal analyses of Hauptmann et al. [5] and Beane Freeman et al. [8] were non-robust and mis-specified as they did not account for an statistically significant interaction structure between plant group (Plant 1 vs. Plants 2–10) and FA exposure (see Models 8 in Additional file 3: Table S3 and Additional file 4: Table S4).

A specific focus of the internal mortality comparisons was to address our concern about the inappropriateness of omitting unexposed workers from the baseline category in exposure-response analyses (Issue 1). We found that analyses using the lowest FA exposure category as the baseline (NCI approach) produced evidence of an exposure-response relationship for FA and NPC for highest peak and average intensity of FA exposure (the basis of NCIs conclusion [8]). In contrast, our corresponding analyses using unexposed workers as the more appropriate baseline category yielded lower RRs for the exposure categories and little or no evidence of an exposure-response association for any of the FA metrics considered. Again, NCI’s finding of only one additional NPC death in the lower FA exposure categories contributed to this null finding.

Our internal analyses also addressed NCI’s practice of mixing results of internal mortality comparisons based on categorical analyses with trend tests based on the continuous form of the FA metric considered. More appropriately, our internal analyses matched the results of the analysis (categorical RRs or slope estimates) with the corresponding trend tests based on categorical or continuous (or pseudo-continuous) scores, respectively. While the p-values associated with these two sets of trend tests differed, in most cases these differences were quantitative and the tests consistently rejected or failed to reject the null hypothesis of no association between FA and NPC.

To address Issue 3, we focused on two aspects of risk analysis to explore a possible mis-specification of the models as presented in Beane Freeman et al. [8], confounder adjustment and interaction assessment. Confounding is understood as defined by Greenland and Robins [43] and as explicated graphically in Greenland et al. [44]. We explored confounding in practice by applying the change-in-estimate criterion [45, 46]. Models 1 and 2 of Additional file 3: Table S3 a, b, c, d gave results about the possible confounding effect of the plant group indicator. Although not pronounced, some indication of confounding was indicted in peak exposure and average intensity models because the relative risk in the highest exposure category decreased after taking the plant group indicator into account. Using the continuous peak exposure variable, the same tendency can be seen as a somewhat reduced risk estimate after adjustment for plant group in the peak exposure model but not so in the other analyses. Therefore, the statement of Hauptmann et al. [5] that the risk estimates for FA exposure did not change considerably after adjusting for plants is confirmed again in this re-analysis. We have observed this in our previous analysis too [23].

Beane Freeman et al. [8] did not perform a risk analysis adjusted for plant or plant group. They performed what they called an “influence analysis” by “excluding one plant at a time”. Such an analysis cannot contrast the findings of Plant 1 vs Plants 2–10 because it does not cover the important case of studying Plant 1 alone. The authors, however, studied Plants 2–10 as a group: “When Plant 1 was excluded, the number of NPC deaths was two in the highest peak exposure category (RR = 3.36, 95 % CI: 0.3, 37.27), one in the highest average intensity category (RR = 4.09, 95 % CI: 0.25, 66.0), and zero in the highest cumulative exposure category.” This can be compared with our findings in Additional file 1: Table S1a-c. The relative estimate is 2.92 (95 % CI: 0.15, 177.22) for the highest peak exposure category, 4.08 (95 % CI: 0.05, 326.39) for the highest average intensity category and 6.74 (95 % CI: 0.32, 428.37) for the highest cumulative exposure category using the low exposure group as the baseline. However, the corresponding relative estimate decreased to 0.43 (95 % CI: 0.02, 7.92) for the highest peak category, 0.42 (95 % CI: 0.01, 9.65) for the highest average intensity category and 0.44 (95 % CI: 0.04, 16.12) for the highest cumulative exposure category with unexposed group as the baseline.

Beane Freeman et al. [8] concluded from their “influence analysis” that they found “. . . *no evidence of plant heterogeneity for a broad group of metrics, including peak exposure.”* We judge that this statement is wrong. We base our judgement on the findings of our interaction analyses (Issue 3). We begin with stating that the full interaction models (Models 9 in Additional file 3: Table S6 a, b, c, d) showed instabilities: The coefficient for the plant group indicator was always accompanied with a lower 95 % CI limit of –infinity. Accordingly, the likelihood ratio p-values were 100 % for the plant group variable in all analyses with the exception of 42 % when analyzing peak exposures. Thus, it is of interest to reduce the models by dropping the plant variable indicator from the Models 9. This means to force the baseline risk of all plants to be the same and then check for different slopes, although usually recommendations are given not to drop main effects if interactions are explored [45]. These reduced models without the main effect of plant group are presented as Models 8 in Additional file 3: Table S3 a, b, c, d. Because the reduced model uses all cases simultaneously (more power than the separate models) and avoids the problem of relying on the very imprecise baseline risk in Plant 1 (disadvantage of the full interaction model), the estimates are more stable: no median unbiased estimates were necessary and no confidence interval limit approached infinity. The interaction terms were found to be significant at the 5 %-level in all analyses (exception: average exposure analysis returned a likelihood ratio p-value of 0.063).

It has been argued to use the p-value of the interaction term in the decision process when assessing interactions [47]. A conservative approach, however, was recommended, i.e., comparing the p-value of the interaction term with a cut point clearly higher than the usual significance level of 5 %: keep the interaction terms within the models if their p-values are not higher than 25 % [45]. This recommendation is in line with the statement that “in epidemiological settings, the power to detect statistical interactions is typically an order of magnitude less than the power to detect main effects” [48]. Following this advice, our re-analyses found clear evidence of an interaction effect of all three FA exposure metrics and the plant group indicator which cannot be ignored.

We conclude from these analyses that there is no NPC risk identified in Plants 2–10 and all effects of formaldehyde that were described in Beane Freeman et al. [8] stem from Plant 1 only. It is curious that Beane Freeman et al. [8] did not follow the advice given in Marsh et al. [23] to perform a regular interaction analysis, but conducted an “influential analysis” (see above). This type of analysis never analyzed Plant 1 alone and was, therefore, unable to judge the degree of heterogeneity between Plant 1 and Plant 2–10. Marsh et al. [11] explained the misinterpretation by Beane Freeman et al. [8] of the previous interaction analyses performed in Marsh et al. [23] and showed that the results presented by Beane Freeman et al. [8] are entirely consistent with the interaction effect observed in Marsh et al. [23].

We emphasize that the current re-analyses strengthen the argument made in Marsh et al. [23] and Marsh et al. [11], that is, we showed a pronounced positive interaction effect (risk modification) by plant group (Plant 1 vs. Plants 2–10), not only for the continuous peak exposure metric but also for average and cumulative exposure and duration of exposure to FA. It follows that the internal modelling approaches presented by Hauptmann et al. [5] were mis-specified and that Beane Freeman et al. [8] did not correct this flaw, but repeated the misleading model set-up.

## Conclusions

The results of the analysis of nasopharyngeal cancer risk in the NCI cohort published by Beane Freeman et al. [8] are misleading because they are based on inappropriate regression analyses. The authors repeatedly failed to account for an important interaction structure between the plant group and the exposure variable which prohibits a generalization of formaldehyde effects within the NCI cohort and, in particular, beyond the NCI cohort. Overall, our updated reanalysis provided little or no evidence to support NCI’s suggestion of a persistent association between FA exposure and mortality from NPC. NCI’s suggestion continues to be driven heavily by anomalous findings in one study plant (Plant 1). Our findings continue to cast considerable additional uncertainty regarding the validity of NCI’s suggested persistent association. This may be of particular interest given the conflicting evaluation of FA carcinogenicity by US and EU authorities.

### Exemptions

This research was deemed exempt from human subjects review by the University of Pittsburgh Institutional Review Board.

## Additional files

Additional file 1: Table S1.
**a** NCI FA cohort, NPC RR results for highest peak FA exposure (ppm) metric, exact estimation. **b** NCI FA cohort, NPC RR results for average intensity of FA exposure (AIE) (ppm) metric, exact estimation. **c** NCI FA cohort, NPC RR results for cumulative FA exposure (Cum) (ppm-years) metric, exact estimation. **d** NCI FA cohort, NPC RR results for duration of FA exposure (Dur) (years) metric, exact estimation. (DOCX 85 kb) Additional file 2: Table S2.
**a** NCI FA cohort, RR analysis using highest peak FA exposure (ppm), asymptotic estimation. **b** NCI FA cohort, RR analysis using average intensity of FA exposure (ppm), asymptotic estimation. **c** NCI FA cohort, RR analysis using cumulative FA exposure (ppm-years), asymptotic estimation. **d** NCI FA cohort, RR analysis using duration of FA exposure (years), asymptotic estimation. (DOCX 54 kb) Additional file 3: Table S3.
**a** Observed deaths and interval rate-based RRs or β coefficients by highest peak FA exposure. **b** Observed deaths and interval rate-based RRs or β coefficients by average intensity of FA exposure (ppm). **c** Observed deaths and interval rate-based RRs or β coefficients by cumulative FA exposure (ppm-years). **d** Observed deaths and interval rate-based RRs or β coefficients by duration of FA exposure (years). (DOCX 77 kb) Additional file 4: Table S4.
**a** Observed deaths and interval RRs or β coefficients by peak FA exposure, asymptotic estimation. **b** Observed deaths and interval RRs or β coefficients by average FA intensity exposure, asymptotic estimation. **c** Observed deaths and RRs or β coefficients by cumulative FA exposure, asymptotic estimation. **d** Observed deaths and RRs or β coefficients by duration of FA exposure, asymptotic estimation. (DOCX 66 kb)

## Footnotes

1 http://echa.europa.eu/web/guest/about-us/who-we-are/committee-for-risk-assessment

2 ECHA, http://echa.europa.eu

3 http://echa.europa.eu/web/guest/view-article/-/journal_content/c89bdb13-09e9-497c-8e73-ddae13a842c8

4 Checkoway et al. [28] redefined the original NCI peak exposure metric in their re-analysis: “at least 1 continuous month of employment in jobs identified in the original exposure characterization as likely having short-term exposure excursions of 2 ppm or more to less than 4 ppm or 4 ppm or more on a weekly or daily basis”. This re-definition, however, had no relevant impact on the results: “our re-analysis using redefined ‘peak’ exposure detected associations similar to those previously reported”. Thus, we did not change the NCI definition and used the highest peak exposure metric as originally applied by NCI [8] and in our previous re-analyses [22, 23, 33].

5 The exact estimation-based RRs based on NCI categories shown in Additional file 1: Table S1a–d differ somewhat from those presented by Beane Freeman et al. [8] who used asymptotic Poisson regression models.
