# Supplementary material for: An updated re-analysis of the mortality risk from nasopharyngeal cancer in the National Cancer Institute formaldehyde worker cohort study
Source: J Occup Med Toxicol. 2016 Mar 2;11:8. doi: 10.1186/s12995-016-0097-6 (PMC4774098; doi:10.1186/s12995-016-0097-6)
Supplement: Additional file 1: Table S1. — a NCI FA cohort, NPC RR results for highest peak FA exposure (ppm) metric, exact estimation. b NCI FA cohort, NPC RR results for average intensity of FA exposure (AIE) (ppm) metric, exact estimation. c NCI FA cohort, NPC RR results for cumulative FA exposure (Cum) (ppm-years) metric, exact estimation. d NCI FA cohort, NPC RR results for duration of FA exposure (Dur) (years) metric, exact estimation. (DOCX 85 kb) [file 12995_2016_97_MOESM1_ESM.docx]

**Table S1a**

**NCI FA cohort, NPC RR results for highest peak FA exposure (ppm) metric ^c,h^, exact estimation**

| **All Plants** | | | | | | | | | | | |  |
| --- | --- | --- | --- | --- | --- | --- | --- | --- | --- | --- | --- | --- |
| **Unexposed Baseline ^i^** | **Obs/df** | **RR** | | **95% CI** | **Low Exposed Baseline ^j^** | **Obs/df** | | **RR** | | **95% CI** | |  |
| NCI Cats.^a,h^  **Unexposed (Baseline)**  Exp Cat 1  Exp Cat 2^d^  Exp Cat 3 | 2  1  0  7 | 1.00  0.25  0.28  1.67 | (0.00,5.39)  (0.00,2.45)  (0.24,19.90) | | NCI Cats.  Unexposed  **Exp Cat 1 (Baseline)**  Exp Cat 2^d^  Exp Cat 3 | 2  1  0  7 | | 4.05  1.00  1.27  7.23 | | (0.19,267.79)  (0.00,24.08)  (0.92,327.62) | |  |
| Slope estimate (Score1^e^)  Trend p-value (Score1)  Slope estimate (Score2 ^f^)  Trend p-value (Score2)  Global p-value | 1  1  3 | 1.68  0.122  1.33  0.131  0.021* | | | Slope estimate (Score1)  Trend p-value (Score1)  Slope estimate (Score2)  Trend p-value (Score2)  Global p-value | 1  1  2 | | 4.22  0.010*  1.76  0.006**  0.006** | | | |  |
| UPitt Cats.^b,h^  **Unexposed (Baseline)**  Exp Cat 1  Exp Cat 2^d^  Exp Cat 3 | 2  1  0  8 | 1.00  0.23  0.26  1.80 | (0.00,5.07)  (0.00,2.30)  (0.28,20.82) | | UPitt Cats.  Unexposed  **Exp Cat 1 (Baseline)**  Exp Cat 2^d^  Exp Cat 3 | 2  1  0  8 | | 4.27  1.00  1.27  8.18 | | (0.20,279.55)  (0.00,24.03)  (1.09,364.94) | |  |
| Slope estimate (Score1)  Trend p-value (Score1)  Slope estimate (Score2)  Trend p-value (Score2)  Global p-value | 1  1  3 | 1.81  0.073  1.37  0.083  0.009** | | | Slope estimate (Score1)  Trend p-value (Score1)  Slope estimate (Score2)  Trend p-value (Score2)  Global p-value | 1  1  2 | | | 4.65  0.004**  1.82  0.004**  0.004** | | | |
| **Plant 1** | | | | | | | | | | | |  |
| **Unexposed Baseline** | **Obs/df** | **RR** | | **95% CI** | **Low Exposed Baseline** | **Obs/df** | | **RR** | | **95% CI** | |  |
| NCI Cats.  **Unexposed (Baseline)**  Exp Cat 1  Exp Cat 2  Exp Cat 3^d^ | 0  0  0  5 | 1.00  NA NA 1.87 | NA NA (0.27,+$\infty)$ | | NCI Cats.  Unexposed  **Exp Cat 1 (Baseline)**  Exp Cat 2  Exp Cat 3^d^ | 0  0  0  5 | | NA  1.00  NA  0.29 | | NA  NA  (0.05,+$\infty)$ | |  |
| Slope estimate (Score1)^d^  Trend p-value (Score1)  Slope estimate (Score2)^d^  Trend p-value (Score2)  Global p-value | 1  1  3 | 5.82  0.040*  1.83  0.083  0.175 | | | Slope estimate (Score1)^d^  Trend p-value (Score1)  Slope estimate (Score2)^d^  Trend p-value (Score2)  Global p-value | 1  1  2 | | 5.76  0.048*  1.80  0.170  0.170 | | | |  |
| UPitt Cats.  **Unexposed (Baseline)**  Exp Cat 1  Exp Cat 2  Exp Cat 3^d^ | 0  0  0  6 | 1.00  NA NA  1.93 | NA NA (0.28,+$\infty)$ | | UPitt Cats.  Unexposed  **Exp Cat 1 (Baseline)**  Exp Cat 2  Exp Cat 3^d^ | 0  0  0  6 | | NA  1.00  NA 0.29 | | NA  NA (0.05,+$\infty)$ | |  |
| Slope estimate (Score1)^d^  Trend p-value (Score1)  Slope estimate (Score2)^d^  Trend p-value (Score2)  Global p-value | 1  1  3 | 6.49  0.026*  1.89  0.054  0.112 | | | Slope estimate (Score1)^d^  Trend p-value (Score1)  Slope estimate (Score2)^d^  Trend p-value (Score2)  Global p-value | 1  1  2 | 6.44  0.048*  1.86  0.108  0.108 | | | |  |  |
| **Plants 2-10** | | | | | | | | | | | |  |
| **Unexposed Baseline** | **Obs/df** | **RR** | | **95% CI** | **Low Exposed Baseline** | **Obs/df** | | **RR** | | **95% CI** | |  |
| NCI Cats.  **Unexposed (Baseline)**  Exp Cat 1  Exp Cat 2^d^  Exp Cat 3 | 2  1  0  2 | 1.00  0.15  0.30  0.43 | (0.00,3.91)  (0.00,2.81)  (0.02,7.92) | | NCI Cats.  Unexposed  **Exp Cat 1 (Baseline)**  Exp Cat 2^d^  Exp Cat 3 | 2  1  0  2 | | 6.62  1.00  2.04  2.92 | | (0.26,488.17)  (0.00,38.78)  (0.15,177.22) | |  |
| Slope estimate (Score1)  Trend p-value (Score1)  Slope estimate (Score2)  Trend p-value (Score2)  Global p-value | 1  1  3 | 0.84  0.829  1.01  0.666  0.242 | | | Slope estimate (Score1)  Trend p-value (Score1)  Slope estimate (Score2)  Trend p-value (Score2)  Global p-value | 1  1  2 | | 2.01  0.516  1.34  0.435  0.435 | | | |  |
| UPitt Cats.  **Unexposed (Baseline)**  Exp Cat 1  Exp Cat 2^d^  Exp Cat 3 | 2  1  0  2 | 1.00  0.15  0.30  0.43 | (0.00,3.91) (0.00,2.81) (0.02,7.92) | | UPitt Cats.  Unexposed  **Exp Cat 1 (Baseline)**  Exp Cat 2^d^  Exp Cat 3 | 2  1  0  2 | | 6.62  1.00  2.04  2.92 | | (0.26,488.17)  (0.00,38.78)  (0.15,177.22) | |  |
| Slope estimate (Score1)  Trend p-value (Score1)  Slope estimate (Score2)  Trend p-value (Score2)  Global p-value | 1  1  3 | 0.84  0.829  1.01  0.666  0.242 | | | Slope estimate (Score1)  Trend p-value (Score1)  Slope estimate (Score2)  Trend p-value (Score2)  Global p-value | 1  1  2 | | 2.01  0.516  1.34  0.435  0.435 | | | |  |

**Table S1b**

**NCI FA cohort, NPC RR results for average intensity of FA exposure (AIE) (ppm) metric ^c,h^, exact estimation**

| **All Plants** | | | | | | | | | |
| --- | --- | --- | --- | --- | --- | --- | --- | --- | --- |
| **Unexposed Baseline ^i^** | **Obs/df** | **RR** | | **95% CI** | | **Low Exposed Baseline ^j^** | **Obs/df** | **RR** | **95% CI** |
| NCI Cats. ^a,h^  **Unexposed (Baseline)**  Exp Cat 1  Exp Cat 2  Exp Cat 3 | 2  1  1  6 | 1.00  0.16  0.40  1.69 | (0.00,3.51)  (0.01,8.72)  (0.24,20.11) | | | NCI Cats.  Unexposed  **Exp Cat 1 (Baseline)**  Exp Cat 2  Exp Cat 3 | 2  1  1  6 | 6.33  1.00  2.54  11.29 | (0.29,422.62)  (0.03,199.55)  (1.35,525.71) |
| Slope estimate (Score1 ^e^)  Trend p-value (Score1)  Slope estimate (Score3 ^g^)  Trend p-value (Score3)  Global p-value | 1  1  3 | 1.80  0.079  4  0.023*  0.024* | | | | Slope estimate (Score1)  Trend p-value (Score1)  Slope estimate (Score3)  Trend p-value (Score3)  Global p-value | 1  1  2 | 3.61  0.005**  10.01  0.01*  0.010* | |
| UPitt Cats. ^b,h^  **Unexposed (Baseline)**  Exp Cat 1  Exp Cat 2  Exp Cat 3 | 2  4  2  3 | 1.00  0.41  1.74  1.42 | (0.05,5.33)  (0.11,26.67)  (0.14,19.51) | | | UPitt Cats.  Unexposed  **Exp Cat 1 (Baseline)**  Exp Cat 2  Exp Cat 3 | 2  4  2  3 | 2.46  1.00  4.18  3.46 | (0.19,21.16)  (0.37,29.58)  (0.50,20.88) |
| Slope estimate (Score1)  Trend p-value (Score1)  Slope estimate (Score3)  Trend p-value (Score3)  Global p-value | 1  1  3 | 1.48  0.209  2.17  0.101  0.101 | | | | Slope estimate (Score1)  Trend p-value (Score1)  Slope estimate (Score3)  Trend p-value (Score3)  Global p-value | 1  1  2 | 1.96  0.056  2.84  0.063  0.063 | |
| **Plant 1** | | | | | | | | | |
| **Unexposed Baseline** | **Obs/df** | **RR** | | **95% CI** | | **Low Exposed Baseline** | **Obs/df** | **RR** | **95% CI** |
| NCI Cats.  **Unexposed (Baseline)**  Exp Cat 1  Exp Cat 2  Exp Cat 3^d^ | 0  0  0  5 | 1.00  NA  NA  1.33 | NA NA (0.19, +$\infty)$ | | | NCI Cats.  Unexposed  **Exp Cat 1 (Baseline)**  Exp Cat 2  Exp Cat 3 | 0  0  0  5 | NA 1.00  NA  0.66 | NA  NA  (0.12,+$\infty)$ |
| Slope estimate (Score1)^d^  Trend p-value (Score1)  Slope estimate (Score3) ^d^  Trend p-value (Score3)  Global p-value | 1  1  3 | 2.69  0.181  6.89  0.551  0.551 | | | Slope estimate (Score1)^d^  Trend p-value (Score1)  Slope estimate (Score3)^d^  Trend p-value (Score3)  Global p-value | | 1  1  2 | 2.50  0.284  5.4  0.536  0.536 | |
| UPitt Cats.  **Unexposed (Baseline)**  Exp Cat 1^d^  Exp Cat 2^d^  Exp Cat 3^d^ | 0  2  2  2 | 1.00  0.80  1.08  0.98 | (0.08,+$\infty)$  (0.12,+$\infty)$  (0.11,+$\infty)$ | | | UPitt Cats.  Unexposed^d^  **Exp Cat 1 (Baseline)**  Exp Cat 2  Exp Cat 3 | 0  2  2  2 | 1.26  1.00  1.25  1.12 | (0.00,11.84)  (0.09,17.49)  (0.08,15.90) |
| Slope estimate (Score1)  Trend p-value (Score1)  Slope estimate (Score3)  Trend p-value (Score3)  Global p-value | 1  1  3 | 1.31  0.547  1.47  1.000  1.000 | | | Slope estimate (Score1)  Trend p-value (Score1)  Slope estimate (Score3)  Trend p-value (Score3)  Global p-value | | 1  1  2 | 1.06  1.000  1.11  1.000  1.000 | |

| **Plants 2-10** | | | | | | | | | | |
| --- | --- | --- | --- | --- | --- | --- | --- | --- | --- | --- |
| **Unexposed Baseline** | **Obs/df** | **RR** | | **95% CI** | | **Low Exposed Baseline** | **Obs/df** | **RR** | **95% CI** | |
| NCI Cats.  **Unexposed (Baseline)**  Exp Cat 1  Exp Cat 2  Exp Cat 3 | 2  1  1  1 | 1.00  0.11  0.37  0.42 | (0.00,2.67)  (0.01,9.08)  (0.01,9.65) | | | NCI Cats.  Unexposed  **Exp Cat 1 (Baseline)**  Exp Cat 2  Exp Cat 3 | 2  1  1  1 | 9.50  1.00  3.59  4.08 | | (0.38,689.85)  (0.05,282.18)  (0.05,326.39) |
| Slope estimate (Score1)  Trend p-value (Score1)  Slope estimate (Score3)  Trend p-value (Score3)  Global p-value | 1  1  3 | 0.89  1.000  1.33  0.121  0.121 | | | | Slope estimate (Score1)  Trend p-value (Score1)  Slope estimate (Score3)  Trend p-value (Score3)  Global p-value | 1  1  2 | 2.15  0.185  3.97  0.245  0.245 | | |
| UPitt Cats.  **Unexposed (Baseline)**  Exp Cat 1  Exp Cat 2^d^  Exp Cat 3 | 2  2  0  1 | 1.00  0.16  1.83  0.63 | (0.01,2.98)  (0.00,16.56)  (0.01,15.59) | | | UPitt Cats.  Unexposed  **Exp Cat 1 (Baseline)**  Exp Cat 2^d^  Exp Cat 3 | 2  2  0  1 | 6.31  1.00  11.58  3.93 | | (0.34,113.60)  (0.00,103.86)  (0.06,79.18) |
| Slope estimate (Score1)  Trend p-value (Score1)  Slope estimate (Score3)  Trend p-value (Score3)  Global p-value | 1  1  3 | 0.87  0.752  1.30  0.193  0.193 | | | Slope estimate (Score1)  Trend p-value (Score1)  Slope estimate (Score3)  Trend p-value (Score3)  Global p-value | | 1  1  2 | 2.34  0.328  3.29  0.328  0.328 | | |

**Table S1c**

**NCI FA cohort, NPC RR results for cumulative FA exposure (Cum) (ppm-years) metric ^c,h^, exact estimation**

| **All Plants** | | | | | | | |
| --- | --- | --- | --- | --- | --- | --- | --- |
| **Unexposed Baseline ^i^** | **Obs/df** | **RR** | **95% CI** | **Low Exposed Baseline ^j^** | **Obs/df** | **RR** | **95% CI** |
| NCI Cats. ^a,h^  **Unexposed (Baseline)**  Exp Cat 1  Exp Cat 2  Exp Cat 3 | 2  4  1  3 | 1.00  0.55  0.53  1.60 | (0.06,7.14)  (0.01,11.30)  (0.14,24.02) | NCI Cats.  Unexposed  **Exp Cat 1 (Baseline)**  Exp Cat 2  Exp Cat 3 | 2  4  1  3 | 1.82  1.00  0.91  2.91 | (0.14,15.66)  (0.02,9.27)  (0.41,17.78) |
| Slope estimate (Score1 ^e^)  Trend p-value (Score1)  Slope estimate (Score3 ^g^)  Trend p-value (Score3)  Global p-value | 1  1  3 | 1.31  0.455  1.1  0.437  0.437 | | Slope estimate (Score1)  Trend p-value (Score1)  Slope estimate (Score3)  Trend p-value (Score3)  Global p-value | 1  1  2 | 1.74  0.162  1.13  0.264  0.264 | |
| UPitt Cats. ^b,h^  **Unexposed (Baseline)**  Exp Cat 1  Exp Cat 2  Exp Cat 3 | 2  4  2  3 | 1.00  0.65  0.49  2.84 | (0.08,8.35)  (0.03,7.64)  (0.25,43.38) | UPitt Cats.  Unexposed  **Exp Cat 1 (Baseline)**  Exp Cat 2  Exp Cat 3 | 2  4  2  3 | 1.55  1.00  0.73  4.37 | (0.12,13.13)  (0.07,5.15)  (0.61,27.29) |
| Slope estimate (Score1)  Trend p-value (Score1)  Slope estimate (Score3)  Trend p-value (Score3)  Global p-value | 1  1  3 | 1.40  0.437  1.10  0.119  0.119 | | Slope estimate (Score1)  Trend p-value (Score1)  Slope estimate (Score3)  Trend p-value (Score3)  Global p-value | 1  1  2 | 2.04  0.126  1.11  0.073  0.073 | |
| **Plant 1** | | | | | | | |
| **Unexposed Baseline** | **Obs/df** | **RR** | **95% CI** | **Low Exposed Baseline** | **Obs/df** | **RR** | **95% CI** |
| NCI Cats.  **Unexposed (Baseline)**  Exp Cat 1^d^  Exp Cat 2^d^  Exp Cat 3^d^ | 0  3  1  1 | 1.00  0.91  0.89  1.22 | (0.12,+$\infty)$  (0.05,+$\infty)$  (0.06,+$\infty)$ | NCI Cats.  Unexposed^d^  **Exp Cat 1 (Baseline)**  Exp Cat 2  Exp Cat 3 | 0  3  1  1 | 1.11  1.00  1.12  2.07 | (0.00,8.61)  (0.02,14.42)  (0.04,30.38) |
| Slope estimate (Score1)  Trend p-value (Score1)  Slope estimate (Score3)  Trend p-value (Score3)  Global p-value | 1  1  3 | 1.65  0.380  1.09  0.711  0.711 | | Slope estimate (Score1)  Trend p-value (Score1)  Slope estimate (Score3)  Trend p-value (Score3)  Global p-value | 1  1  2 | 1.39  0.525  1.08  0.78  0.780 | |
| UPitt Cats.  **Unexposed (Baseline)**  Exp Cat 1^d^  Exp Cat 2^d^  Exp Cat 3^d^ | 0  3  2  1 | 1.00  1.06  0.80  2.67 | (0.14,+$\infty)$  (0.09,+$\infty)$  (0.14,+$\infty)$ | UPitt Cats.  Unexposed^d^  **Exp Cat 1 (Baseline)**  Exp Cat 2  Exp Cat 3 | 0  3  2  1 | 0.94  1.00  0.83  3.87 | (0.00,7.22)  (0.07,7.45)  (0.07,57.48) |
| Slope estimate (Score1)  Trend p-value (Score1)  Slope estimate (Score3)  Trend p-value (Score3)  Global p-value | 1  1  3 | 1.84  0.374  1.10  0.422  0.422 | | Slope estimate (Score1)  Trend p-value (Score1)  Slope estimate (Score3)  Trend p-value (Score3)  Global p-value | 1  1  2 | 1.50  0.502  1.09  0.329  0.329 | |

| **Plants 2-10** | | | | | | | |
| --- | --- | --- | --- | --- | --- | --- | --- |
| **Unexposed Baseline** | **Obs/df** | **RR** | **95% CI** | **Low Exposed Baseline** | **Obs/df** | **RR** | **95% CI** |
| NCI Cats.  **Unexposed (Baseline)**  Exp Cat 1  Exp Cat 2^d^  Exp Cat 3 | 2  1  0  2 | 1.00  0.11  0.40  0.77 | (0.00,2.93)  (0.00,3.70)  (0.04,16.12) | NCI Cats.  Unexposed  **Exp Cat 1 (Baseline)**  Exp Cat 2^d^  Exp Cat 3 | 2  1  0  2 | 8.75  1.00  3.56  6.74 | (0.34,642.96)  (0.00,67.64)  (0.32,428.37) |
| Slope estimate (Score1)  Trend p-value (Score1)  Slope estimate (Score3)  Trend p-value (Score3)  Global p-value | 1  1  3 | 1.11  0.799  1.13  0.111  0.111 | | Slope estimate (Score1)  Trend p-value (Score1)  Slope estimate (Score3)  Trend p-value (Score3)  Global p-value | 1  1  2 | 3.51  0.093  1.3  0.1  0.100 | |
| UPitt Cats.  **Unexposed (Baseline)**  Exp Cat 1  Exp Cat 2^d^  Exp Cat 3 | 2  1  0  2 | 0.14  1.00  0.21  1.33 | (0.00,3.67)  (0.00,1.93)  (0.07,29.17) | UPitt Cats.  Unexposed  **Exp Cat 1 (Baseline)**  Exp Cat 2^d^  Exp Cat 3 | 2  1  0  2 | 7.23  1.00  1.51  9.45 | (0.27,543.59)  (0.00,28.78)  (0.43,619.36) |
| Slope estimate (Score1)  Trend p-value (Score1)  Slope estimate (Score3)  Trend p-value (Score3)  Global p-value | 1  1  3 | 1.08  1.000  1.13  0.023*  0.023* | | Slope estimate (Score1)  Trend p-value (Score1)  Slope estimate (Score3)  Trend p-value (Score3)  Global p-value | 1  1  2 | 5.01  0.070  1.23  0.027*  0.027* | |

**Table S1d**

**NCI FA cohort, NPC RR results for duration of FA exposure (Dur) (years) metric ^c,h^, exact estimation**

| **All Plants** | | | | | | | |
| --- | --- | --- | --- | --- | --- | --- | --- |
| **Unexposed Baseline ^i^** | **Obs/df** | **RR** | **95% CI** | **Low Exposed Baseline ^j^** | **Obs/df** | **RR** | **95% CI** |
| NCI Cats. ^a,h^  **Unexposed (Baseline)**  Exp Cat 1  Exp Cat 2  Exp Cat 3 | 2  5  0  3 | 1.00  0.67  0.51  1.72 | (0.09,8.30)  (0.01,11.13)  (0.08,39.22) | NCI Cats.  Unexposed  **Exp Cat 1 (Baseline)**  Exp Cat 2  Exp Cat 3 | 2  5  0  3 | 1.49  1.00  0.73  2.55 | (0.12,11.50)  (0.02,6.58)  (0.20,22.24) |
| Slope estimate (Score1 ^e^)  Trend p-value (Score1)  Slope estimate (Score3 ^g^)  Trend p-value (Score3)  Global p-value | 1  1  3 | 1.17  0.677  1.04  0.669  0.669 | | Slope estimate (Score1)  Trend p-value (Score1)  Slope estimate (Score3)  Trend p-value (Score3)  Global p-value | 1  1  2 | 1.45  0.438  1.04  0.449  0.449 | |
| UPitt Cats. ^b,h^  **Unexposed (Baseline)**  Exp Cat 1  Exp Cat 2  Exp Cat 3 | 2  3  3  3 | 1.00  0.69  0.69  0.88 | (0.07,9.65) (0.07,9.24)  (0.08,13.32) | UPitt Cats.  Unexposed  **Exp Cat 1 (Baseline)**  Exp Cat 2  Exp Cat 3 | 2  3  3  3 | 1.45  1.00  0.99  1.28 | (0.10,15.10)  (0.13,7.48)  (0.17,9.85) |
| Slope estimate (Score1)  Trend p-value (Score1)  Slope estimate (Score3)  Trend p-value (Score3)  Global p-value | 1  1  3 | 0.99  1.000  1.01  0.974  0.974 | | Slope estimate (Score1)  Trend p-value (Score1)  Slope estimate (Score3)  Trend p-value (Score3)  Global p-value | 1  1  2 | 1.15  0.832  1.03  1.000  1.000 | |
| **Plant 1** | | | | | | | |
| **Unexposed Baseline** | **Obs/df** | **RR** | **95% CI** | **Low Exposed Baseline** | **Obs/df** | **RR** | **95% CI** |
| NCI Cats.  **Unexposed (Baseline)**  Exp Cat 1^d^  Exp Cat 2  Exp Cat 3^d^ | 0  4  0  1 | 1.00  1.07  NA  3.86 | (0.15,+$\infty)$  NA  (0.20,+$\infty)$ | NCI Cats.  Unexposed^d^  **Exp Cat 1 (Baseline)**  Exp Cat 2^d^  Exp Cat 3 | 0  4  0  1 | 0.93  1.00  1.49  8.78 | (0.00,6.68)  (0.00,9.48)  (0.11,691.02) |
| Slope estimate (Score1)  Trend p-value (Score1)  Slope estimate (Score3)  Trend p-value (Score3)  Global p-value | 1  1  3 | 2.44  0.209  1.09  0.32  0.320 | | Slope estimate (Score1)  Trend p-value (Score1)  Slope estimate (Score3)  Trend p-value (Score3)  Global p-value | 1  1  2 | 2.08  0.258  1.09  0.258  0.258 | |
| UPitt Cats.  **Unexposed (Baseline)**  Exp Cat 1^d^  Exp Cat 2^d^  Exp Cat 3^d^ | 0  3  2  1 | 1.00  1.01  0.95  0.84 | (0.13,+$\infty)$  (0.10,+$\infty)$  (0.04,+$\infty)$ | UPitt Cats.  Unexposed^d^  **Exp Cat 1 (Baseline)**  Exp Cat 2  Exp Cat 3 | 0  3  2  1 | 0.99  1.00  0.98  1.19 | (0.00,7.65)  (0.08,8.63)  (0.02,16.31) |
| Slope estimate (Score1)  Trend p-value (Score1)  Slope estimate (Score3)  Trend p-value (Score3)  Global p-value | 1  1  3 | 1.33  0.616  1.03  1.000  1.000 | | Slope estimate (Score1)  Trend p-value (Score1)  Slope estimate (Score3)  Trend p-value (Score3)  Global p-value | 1  1  2 | 1.06  1.000  1.01  1.000  1.000 | |

| **Plants 2-10** | | | | | | | |
| --- | --- | --- | --- | --- | --- | --- | --- |
| **Unexposed Baseline** | **Obs/df** | **RR** | **95% CI** | **Low Exposed Baseline** | **Obs/df** | **RR** | **95% CI** |
| NCI Cats.  **Unexposed (Baseline)**  Exp Cat 1  Exp Cat 2  Exp Cat 3 | 2  1  0  2 | 1.00  0.11  0.36  0.43 | (0.00,2.89)  (0.01,8.81)  (0.01,18.39) | NCI Cats.  Unexposed  **Exp Cat 1 (Baseline)**  Exp Cat 2  Exp Cat 3 | 2  1  0  2 | 8.77  1.00  3.13  3.19 | (0.35,639.51)  (0.04,251.78)  (0.04,280.56) |
| Slope estimate (Score1)  Trend p-value (Score1)  Slope estimate (Score3)  Trend p-value (Score3)  Global p-value | 1  1  3 | 0.87  1.000  1.03  0.181  0.181 | | Slope estimate (Score1)  Trend p-value (Score1)  Slope estimate (Score3)  Trend p-value (Score3)  Global p-value | 1  1  2 | 1.79  0.274  1.05  0.323  0.323 | |
| UPitt Cats.  **Unexposed (Baseline)**  Exp Cat 1^d^  Exp Cat 2  Exp Cat 3 | 2  0  1  2 | 1.00  0.23  0.21  0.42 | (0.00,2.14) (0.00,5.03) (0.02,8.77) | UPitt Cats.  Unexposed^d^  **Exp Cat 1 (Baseline)**  Exp Cat 2^d^  Exp Cat 3^d^ | 2  0  1  2 | 4.38  1.00  0.91  2.08 | (0.47,+$\infty)$  (0.05,+$\infty)$  (0.24,+$\infty)$ |
| Slope estimate (Score1)  Trend p-value (Score1)  Slope estimate (Score3)  Trend p-value (Score3)  Global p-value | 1  1  3 | 0.89  0.824  1.04  0.299  0.299 | | Slope estimate (Score1)  Trend p-value (Score1)  Slope estimate (Score3)  Trend p-value (Score3)  Global p-value | 1  1  2 | 3.51  0.281  1.13  0.766  0.766 | |

1. NCI categories based on 60^th^ and 80^th^ percentiles of FA exposure among cancer deaths who were exposed. Includes on 10/11 deaths.
2. UPitt categories based on approximate tertiles of FA exposure among NPC deaths who were exposed. Includes 11 deaths.
3. All exposures lagged 15 years as in NCI study
4. Median unbiased estimator from exact conditional logistic model
5. **Score1:** Assign 1,2,3,4 to the non-exposure, low, median and high exposure groups and treat the exposure as continuous in the model.
6. **Score2:** Pseudo-continuous PEAK score defined as the arithmetic mean of the peak interval, including a reasonable assumption about the score for the last open-ended interval (PEAK score : unexposed=0, >0-1.9=0.95, 2.0-3.9=3.0, 4.0+=6.0)
7. **Score3:** Continuous AIE, CUM and DUR score defined as the median value of each of the NCI/Upitt categories taken from Marsh et al. (2004)

**NCI:** AIE (No exposure=0, >0-<0.5=0.12, 0.5-<1.0=0.7, 1.0+=1.24); CUM (No exposure=0, >0-<1.5=0.2, 1.5-<5.5 =2.72, 5.5+=10.23); DUR (No exposure=0, >0-<5.0=0.61, 5.0-<15.0 =8.94, 15.0+ =21.44);

**Upitt:** AIE (No exposure=0, >0-< 1.046=.21, 1.046-<1.178=1.10, 1.178+=1.55); CUM (No exposure=0, >0-<0.734=0.14, 0.734-<10.151=2.36, 10.151+=16.34); DUR (No exposure=0, >0-<0.617=0.25, 0.617-<6.264=2, 6.264+=13.01).

1. **NCI exposure category cut points:** Highest peak (>0-1.9, 2.0-3.9, 4.0+ ppm); AIE (>0-<0.5, 0.5-<1.0, 1.0+ ppm); CUM(>0-<1.5, 1.5-<5.5, 5.5+ ppm-years); DUR (>0-<5.0, 5.0-<15.0, 15.0+ years), **UPitt exposure category cut points:** Highest peak same as NCI; AIE (>0-< 1.046, 1.046-<1.178, 1.178+ ppm); CUM (>0-<0.734, 0.734-<10.151, 10.151+ ppm-years); DUR (>0-<0.617, 0.617-<6.264, 6.264+ years)
2. All trend test and global test for exposure are among unexposed and exposed workers
3. All trend test and global test for exposure are among exposed workers

*p < 0.05 **p<0.01
