# Supplementary material for: An updated re-analysis of the mortality risk from nasopharyngeal cancer in the National Cancer Institute formaldehyde worker cohort study
Source: J Occup Med Toxicol. 2016 Mar 2;11:8. doi: 10.1186/s12995-016-0097-6 (PMC4774098; doi:10.1186/s12995-016-0097-6)
Supplement: Additional file 2: Table S2. — a NCI FA cohort, RR analysis using highest peak FA exposure (ppm), asymptotic estimation. b NCI FA cohort, RR analysis using average intensity of FA exposure (ppm), asymptotic estimation. c NCI FA cohort, RR analysis using cumulative FA exposure (ppm-years), asymptotic estimation. d NCI FA cohort, RR analysis using duration of FA exposure (years), asymptotic estimation. (DOCX 54 kb) [file 12995_2016_97_MOESM2_ESM.docx]

**Supplementary Table S2-a**

**NCI FA cohort, RR analysis using highest peak FA exposure (ppm) ^c,g^, asymptotic estimation**

| **All Plants** | | | | | | | |
| --- | --- | --- | --- | --- | --- | --- | --- |
| **Unexposed Baseline^h^** | **Obs** | **RR** | **95% CI** | **Low Exposed Baseline^i^** | **Obs** | **RR** | **95% CI** |
| NCI Cats.^a,g^  **Unexposed (Baseline)**  Exp Cat 1  Exp Cat 2  Exp Cat 3 | 2  1  0  7 | 1.00  0.23  0  1.66 | (0.02,2.88)  NA  (0.29,9.62) | NCI Cats.  Unexposed  **Exp Cat 1 (Baseline)**  Exp Cat 2  Exp Cat 3 | 2  1  0  7 | 4.35  1.00  0  7.23 | (0.35-54.40)  NA  (0.88-59.06) |
| Slope estimate (Score1^d^)  Trend p-value (Score1)  Slope estimate (Score2^e^)  Trend p-value (Score2)  Global p-value | 1  1  3 | 1.68  0.108  1.33  0.041*  0.013* | | Slope estimate (Score1)  Trend p-value (Score1)  Slope estimate (Score2)  Trend p-value (Score2)  Global p-value | 1  1  2 | 4.22  0.006**  1.76  0.004**  0.005** | |
| UPitt Cats.^b,g^  **Unexposed (Baseline)**  Exp Cat 1  Exp Cat 2  Exp Cat 3 | 2  1  0  8 | 1.00  0.22  0  1.80 | (0.02,2.72)  NA  (0.32,10.04) | UPitt Cats.  Unexposed  **Exp Cat 1 (Baseline)**  Exp Cat 2  Exp Cat 3 | 2  1  0  8 | 4.55  1.00  0  8.18 | (0.37,56.36)  NA  (1.02,65.74) |
| Slope estimate (Score1)  Trend p-value (Score1)  Slope estimate (Score2)  Trend p-value (Score2)  Global p-value | 1  1  3 | 1.81  0.060  1.37  0.020*  0.006** | | Slope estimate (Score1)  Trend p-value (Score1)  Slope estimate (Score2)  Trend p-value (Score2)  Global p-value | 1  1  2 | 4.66  0.003**  1.82  0.002**  0.002** | |
| **Plant 1** | | | | | | | |
| **Unexposed Baseline** | **Obs** | **RR** | **95% CI** | **Low Exposed Baseline** | **Obs** | **RR** | **95% CI** |
| NCI Cats. (N.C.^j^) Wald^k^  **Unexposed (Baseline)**  Exp Cat 1  Exp Cat 2  Exp Cat 3 | 0  0  0  5 | 1.00  2.1E+120  40.53  8.6E+137 | NA  NA  NA | NCI Cats. (N.C.) Wald  Unexposed  **Exp Cat 1 (Baseline)**  Exp Cat 2  Exp Cat 3 | 0  0  0  5 | 3.6E+108  1.00  0  6.8E+124 | NA  NA  NA |
| Slope estimate (Score1)  Trend p-value (Score1)  Slope estimate (Score2)  Trend p-value (Score2)  Global p-value | 1  1  3 | NA  NA  NA  NA  1.000 | | Slope estimate (Score1)  Trend p-value (Score1)  Slope estimate (Score2)  Trend p-value (Score2)  Global p-value | 1  1  2 | NA  1.000  8.27E+27  1.000  1.000 | |
| UPitt Cats. (N.C.) Wald  **Unexposed (Baseline)**  Exp Cat 1  Exp Cat 2  Exp Cat 3 | 0  0  0  6 | 1.00  1.5E+112 245465.9  3.00E+126 | NA  NA  NA | UPitt Cats. (N.C.) Wald  Unexposed  **Exp Cat 1 (Baseline)**  Exp Cat 2  Exp Cat 3 | 0  0  0  6 | 4.1E+111  1.00  2.40  2.7E+127 | NA  NA  NA |
| Slope estimate (Score1)  Trend p-value (Score1)  Slope estimate (Score2)  Trend p-value (Score2)  Global p-value | 1  1  3 | NA  NA  339933.1  1.000  1.000 | | Slope estimate (Score1)  Trend p-value (Score1)  Slope estimate (Score2)  Trend p-value (Score2)  Global p-value | 1  1  2 | NA  1.000  9.68E+25  1.000  NA | |
| **Plants 2-10** | | | | | | | |
| **Unexposed Baseline** | **Obs** | **RR** | **95% CI** | **Low Exposed Baseline** | **Obs** | **RR** | **95% CI** |
| NCI Cats.  **Unexposed (Baseline)**  Exp Cat 1  Exp Cat 2  Exp Cat 3 | 2  1  0  2 | 1.00  0.15  0  0.42 | (0.01,1.97)  NA  (0.05,3.75) | NCI Cats.  Unexposed  **Exp Cat 1 (Baseline)**  Exp Cat 2  Exp Cat 3 | 2  1  0  2 | 6.88  1.00  0  2.91 | (0.51,93.46)  NA  (0.26,32.87) |
| Slope estimate (Score1)  Trend p-value (Score1)  Slope estimate (Score2)  Trend p-value (Score2)  Global p-value | 1  1  3 | 0.84  0.703  1.01  0.972  0.221 | | Slope estimate (Score1)  Trend p-value (Score1)  Slope estimate (Score2)  Trend p-value (Score2)  Global p-value | 1  1  2 | 2.02  0.300  1.34  0.269  0.305 | |
| UPitt Cats.  **Unexposed (Baseline)**  Exp Cat 1  Exp Cat 2  Exp Cat 3 | 2  1  0  2 | 1.00  0.15  0  0.42 | (0.01,1.97)  NA  (0.05,3.75) | UPitt Cats.  Unexposed  **Exp Cat 1 (Baseline)**  Exp Cat 2  Exp Cat 3 | 2  1  0  2 | 6.88  1.00  0  2.91 | (0.51,93.46)  NA  (0.26,32.87) |
| Slope estimate (Score1)  Trend p-value (Score1)  Slope estimate (Score2)  Trend p-value (Score2)  Global p-value | 1  1  3 | 0.84  0.703  1.01  0.972  0.221 | | Slope estimate (Score1)  Trend p-value (Score1)  Slope estimate (Score2)  Trend p-value (Score2)  Global p-value | 1  1  2 | 2.02  0.300  1.34  0.269  0.305 | |

**Supplementary Table S2-b**

**NCI FA cohort, RR analysis using average intensity of FA exposure (ppm) ^c,g^, asymptotic estimation**

| **All Plants** | | | | | | | |
| --- | --- | --- | --- | --- | --- | --- | --- |
| **Unexposed Baseline^h^** | **Obs** | **RR** | **95% CI** | **Low Exposed Baseline^i^** | **Obs** | **RR** | **95% CI** |
| NCI Cats.**^a,g^**  **Unexposed (Baseline)**  Exp Cat 1  Exp Cat 2  Exp Cat 3 | 2  1  1  6 | 1.00  0.15  0.38  1.68 | (0.01,1.88)  (0.03,4.69)  (0.29,9.67) | NCI Cats.  Unexposed  **Exp Cat 1 (Baseline)**  Exp Cat 2  Exp Cat 3 | 2  1  1  6 | 6.73  1.00  2.53  11.29 | (0.53-85.19)  (0.16-40.52)  (1.34-94.84) |
| Slope estimate (Score1^d^)  Trend p-value (Score1)  Slope estimate (Score4^f^)  Trend p-value (Score4)  Global p-value | 1  1  3 | 1.80  0.063  1.61  0.145  0.042* | | Slope estimate (Score1)  Trend p-value (Score1)  Slope estimate (Score4)  Trend p-value (Score4)  Global p-value | 1  1  2 | 3.61  0.004**  1.86  0.075  0.017* | |
| UPitt Cats. **^b,g^**  **Unexposed (Baseline)**  Exp Cat 1  Exp Cat 2  Exp Cat 3 | 2  4  2  3 | 1.00  0.40  1.67  1.39 | (0.06,2.58)  (0.21,13.17)  (0.20,9.50) | UPitt Cats.  Unexposed  **Exp Cat 1 (Baseline)**  Exp Cat 2  Exp Cat 3 | 2  4  2  3 | 2.49  1.00  4.16  3.46 | (0.39,15.98)  (0.75,22.98)  (0.76,15.74) |
| Slope estimate (Score1)  Trend p-value (Score1)  Slope estimate (Score4)  Trend p-value (Score4)  Global p-value | 1  1  3 | 1.48  0.225  1.69  0.087  0.277 | | Slope estimate (Score1)  Trend p-value (Score1)  Slope estimate (Score4)  Trend p-value (Score4)  Global p-value | 1  1  2 | 1.96  0.078  1.93  0.044*  0.147 | |
| **Plant 1** | | | | | | | |
| **Unexposed Baseline** | **Obs** | **RR** | **95% CI** | **Low Exposed Baseline** | **Obs** | **RR** | **95% CI** |
| NCI Cats. (N.C.^j^) Wald^k^  **Unexposed (Baseline)**  Exp Cat 1  Exp Cat 2  Exp Cat 3 | 0  0  0  5 | 1.00  5.2E+177  0  1.4E+194 | NA  NA  NA | NCI Cats. (N.C.) Wald  Unexposed  **Exp Cat 1 (Baseline)**  Exp Cat 2  Exp Cat 3 | 0  0  0  5 | 0  1.00  0  2.0E+182 | NA  NA  NA |
| Slope estimate (Score1)  Trend p-value (Score1)  Slope estimate (Score4)  Trend p-value (Score4)  Global p-value | 1  1  3 | NA  NA  4.03  0.304  1.000 | | Slope estimate (Score1)  Trend p-value (Score1)  Slope estimate (Score4)  Trend p-value (Score4)  Global p-value | 1  1  2 | NA  NA  2.42  0.587  NA | |
| UPitt Cats. Wald  **Unexposed (Baseline)**  Exp Cat 1  Exp Cat 2  Exp Cat 3 | 0  2  2  2 | 1.00  9.02E+6  1.12E+7  1.01E+7 | NA NA NA | UPitt Cats. Wald  Unexposed  **Exp Cat 1 (Baseline)**  Exp Cat 2  Exp Cat 3 | 0  2  2  2 | 0  1.00  1.24  1.12 | NA  (0.17,8.96)  (0.15,8.15) |
| Slope estimate (Score1)  Trend p-value (Score1)  Slope estimate (Score4)  Trend p-value (Score4)  Global p-value | 1  1  3 | 1.31  0.524  9.85  0.105  0.997 | | Slope estimate (Score1)  Trend p-value (Score1)  Slope estimate (Score4)  Trend p-value (Score4)  Global p-value | 1  1  2 | 1.06  0.909  8.28  0.172  0.977 | |

| **Plants 2-10** | | | | | | | | | | |
| --- | --- | --- | --- | --- | --- | --- | --- | --- | --- | --- |
| **Unexposed Baseline** | **Obs** | | **RR** | **95% CI** | **Low Exposed Baseline** | **Obs** | **RR** | | **95% CI** | |
| NCI Cats.  **Unexposed (Baseline)**  Exp Cat 1  Exp Cat 2  Exp Cat 3 | | 2  1  1  1 | 1.00  0.10  0.36  0.40 | (0.01,1.36)  (0.03,4.73)  (0.03,5.07) | NCI Cats.  Unexposed  **Exp Cat 1 (Baseline)**  Exp Cat 2  Exp Cat 3 | 2  1  1  1 | | 9.98  1.00  3.56  4.02 | | (0.74,134.87)  (0.22,57.15)  (0.25,65.77) |
| Slope estimate (Score1)  Trend p-value (Score1)  Slope estimate (Score4)  Trend p-value (Score4)  Global p-value | | 1  1  3 | 0.89  0.816  1.50  0.364  0.343 | | Slope estimate (Score1)  Trend p-value (Score1)  Slope estimate (Score4)  Trend p-value (Score4)  Global p-value | 1  1  2 | 2.16  0.257  2.47  0.088  0.469 | | | |
| UPitt Cats.  **Unexposed (Baseline)**  Exp Cat 1  Exp Cat 2  Exp Cat 3 | | 2  2  0  1 | 1.00  0.16  0  0.61 | (0.02,1.42)  NA (0.05,8.09) | UPitt Cats.  Unexposed  **Exp Cat 1 (Baseline)**  Exp Cat 2  Exp Cat 3 | 2  2  0  1 | | 6.38  1.00  0  3.89 | | (0.71,57.71)  NA (0.34,45.05) |
| Slope estimate (Score1)  Trend p-value (Score1)  Slope estimate (Score4)  Trend p-value (Score4)  Global p-value | | 1  1  3 | 0.87  0.828  1.50  0.364  0.354 | | Slope estimate (Score1)  Trend p-value (Score1)  Slope estimate (Score4)  Trend p-value (Score4)  Global p-value | 1  1  2 | 2.34  0.252  2.47  0.088  0.414 | | | |

**Supplementary Table S2-c**

**NCI FA cohort, RR analysis using cumulative FA exposure (ppm-years) ^c,g^, asymptotic estimation**

| **All Plants** | | | | | | | | |
| --- | --- | --- | --- | --- | --- | --- | --- | --- |
| **Unexposed Baseline^h^** | **Obs** | **RR** | **95% CI** | **Low Exposed Baseline^i^** | **Obs** | **RR** | | **95% CI** |
| NCI Cats.^a,g^  **Unexposed (Baseline)**  Exp Cat 1  Exp Cat 2  Exp Cat 3 | 2  4  1  3 | 1.00  0.54  0.49  1.57 | (0.08,3.44)  (0.04,6.04)  (0.21,11.75) | NCI Cats.  Unexposed  **Exp Cat 1 (Baseline)**  Exp Cat 2  Exp Cat 3 | 2  4  1  3 | 1.86  1.00  0.91  2.92 | | (0.29-11.84)  (0.10-8.16)  (0.63-13.44) |
| Slope estimate (Score1^d^)  Trend p-value (Score1)  Slope estimate (Score4^f^)  Trend p-value (Score4)  Global p-value | 1  1  3 | 1.31  0.443  1.03  0.112  0.548 | | Slope estimate (Score1)  Trend p-value (Score1)  Slope estimate (Score4)  Trend p-value (Score4)  Global p-value | 1  1  2 | 1.74  0.196  1.04  0.099  0.347 | | |
| UPitt Cats.^b,g^  **Unexposed (Baseline)**  Exp Cat 1  Exp Cat 2  Exp Cat 3 | 2  4  2  3 | 1.00  0.63  0.46  2.80 | (0.10,4.02)  (0.06,3.75)  (0.37,21.25) | UPitt Cats.  Unexposed  **Exp Cat 1 (Baseline)**  Exp Cat 2  Exp Cat 3 | 2  4  2  3 | 1.58  1.00  0.73  4.41 | | (0.25,9.98)  (0.13,4.01)  (0.94,20.69) |
| Slope estimate (Score1)  Trend p-value (Score1)  Slope estimate (Score4)  Trend p-value (Score4)  Global p-value | 1  1  3 | 1.39  0.382  1.04  0.091  0.243 | | Slope estimate (Score1)  Trend p-value (Score1)  Slope estimate (Score4)  Trend p-value (Score4)  Global p-value | 1  1  2 | | 2.04  0.137  1.04  0.081  0.119 | |
| **Plant 1** | | | | | | | | |
| **Unexposed Baseline** | **Obs** | **RR** | **95% CI** | **Low Exposed Baseline** | **Obs** | **RR** | | **95% CI** |
| NCI Cats. Wald^k^  **Unexposed (Baseline)**  Exp Cat 1  Exp Cat 2  Exp Cat 3 | 0  3  1  1 | 1.00  9.17E+6  1.03E+7  1.92E+7 | NA NA NA | NCI Cats. Wald  Unexposed  **Exp Cat 1 (Baseline)**  Exp Cat 2  Exp Cat 3 | 0  3  1  1 | 0  1.00  1.13  2.09 | | NA  (0.11,11.07)  (0.20,22.20) |
| Slope estimate (Score1)  Trend p-value (Score1)  Slope estimate (Score4)  Trend p-value (Score4)  Global p-value | 1  1  3 | 1.65  0.342  1.15  0.055  0.945 | | Slope estimate (Score1)  Trend p-value (Score1)  Slope estimate (Score4)  Trend p-value (Score4)  Global p-value | 1  1  2 | | 1.39  0.578  1.14  0.069  0.828 | |
| UPitt Cats. Wald  **Unexposed (Baseline)**  Exp Cat 1  Exp Cat 2  Exp Cat 3 | 0  3  2  1 | 1.00  9.66E+6  8.06E+6  3.74E+7 | NA  NA  NA | UPitt Cats. Wald  Unexposed  **Exp Cat 1 (Baseline)**  Exp Cat 2  Exp Cat 3 | 0  3  2  1 | 0  1.00  0.83  3.87 | | NA  (0.14,5.10)  (0.36,41.42) |
| Slope estimate (Score1)  Trend p-value (Score1)  Slope estimate (Score4)  Trend p-value (Score4)  Global p-value | 1  1  3 | 1.84  0.298  1.16  0.018*  0.662 | | Slope estimate (Score1)  Trend p-value (Score1)  Slope estimate (Score4)  Trend p-value (Score4)  Global p-value | 1  1  2 | | 1.50  0.541  1.15  0.024*  0.452 | |

| **Plants 2-10** | | | | | | | |
| --- | --- | --- | --- | --- | --- | --- | --- |
| **Unexposed Baseline** | **Obs** | **RR** | **95% CI** | **Low Exposed Baseline** | **Obs** | **RR** | **95% CI** |
| NCI Cats.  **Unexposed (Baseline)**  Exp Cat 1  Exp Cat 2  Exp Cat 3 | 2  1  0  2 | 1.00  0.11  0  0.77 | (0.01,1.49)  NA (0.08,7.51) | NCI Cats.  Unexposed  **Exp Cat 1 (Baseline)**  Exp Cat 2  Exp Cat 3 | 2  1  0  2 | 9.09  1.00  0  6.98 | (0.67,122.93)  NA (0.58,83.41) |
| Slope estimate (Score1)  Trend p-value (Score1)  Slope estimate (Score4)  Trend p-value (Score4)  Global p-value | 1  1  3 | 1.11  0.840  1.04  0.151  0.120 | | Slope estimate (Score1)  Trend p-value (Score1)  Slope estimate (Score4)  Trend p-value (Score4)  Global p-value | 1  1  2 | 3.51  0.088  1.04  0.096  0.106 | |
| UPitt Cats.  **Unexposed (Baseline)**  Exp Cat 1  Exp Cat 2  Exp Cat 3 | 2  1  0  2 | 1.00  0.13  0  1.33 | (0.01,1.85)  NA  (0.13,13.54) | UPitt Cats.  Unexposed  **Exp Cat 1 (Baseline)**  Exp Cat 2  Exp Cat 3 | 2  1  0  2 | 7.45  1.00  0  9.91 | (0.54,102.54)  NA  (0.81,121.70) |
| Slope estimate (Score1)  Trend p-value (Score1)  Slope estimate (Score4)  Trend p-value (Score4)  Global p-value | 1  1  3 | 1.08  0.889  1.04  0.151  0.041* | | Slope estimate (Score1)  Trend p-value (Score1)  Slope estimate (Score4)  Trend p-value (Score4)  Global p-value | 1  1  2 | 5.06  0.060  1.04  0.096  0.030* | |

**Supplementary Table S2-d**

**NCI FA cohort, RR analysis using duration of FA exposure (years) ^c,g^, asymptotic estimation**

| **All Plants** | | | | | | | |
| --- | --- | --- | --- | --- | --- | --- | --- |
| **Unexposed Baseline^h^** | **Obs** | **RR** | **95% CI** | **Low Exposed Baseline^i^** | **Obs** | **RR** | **95% CI** |
| NCI Cats. **^a,g^**  **Unexposed (Baseline)**  Exp Cat 1  Exp Cat 2  Exp Cat 3 | 2  5  1  2 | 1.00  0.66  0.49  1.74 | (0.11,4.02)  (0.04,6.01)  (0.15,19.72) | NCI Cats.  Unexposed  **Exp Cat 1 (Baseline)**  Exp Cat 2  Exp Cat 3 | 2  5  1  2 | 1.49  1.00  0.73  2.62 | (0.25,9.03)  (0.08-6.30)  (0.41-16.53) |
| Slope estimate (Score1^d^)  Trend p-value (Score1^f^)  Slope estimate (Score4)  Trend p-value (Score4)  Global p-value | 1  1  3 | 1.17  0.708  1.03  0.537  0.713 | | Slope estimate (Score1)  Trend p-value (Score1)  Slope estimate (Score4)  Trend p-value (Score4)  Global p-value | 1  1  2 | 1.45  0.446  1.03  0.477  0.601 | |
| UPitt Cats. **^b,g^**  **Unexposed (Baseline)**  Exp Cat 1  Exp Cat 2  Exp Cat 3 | 2  3  3  3 | 1.00  0.68  0.67  0.87 | (0.10,4.68)  (0.10,4.46)  (0.12,6.52) | UPitt Cats.  Unexposed  **Exp Cat 1 (Baseline)**  Exp Cat 2  Exp Cat 3 | 2  3  3  3 | 1.48  1.00  0.99  1.29 | (0.21,10.26)  (0.20,4.94)  (0.25,6.59) |
| Slope estimate (Score1)  Trend p-value (Score1)  Slope estimate (Score4)  Trend p-value (Score4)  Global p-value | 1  1  3 | 0.99  0.979  1.03  0.530  0.966 | | Slope estimate (Score1)  Trend p-value (Score1)  Slope estimate (Score4)  Trend p-value (Score4)  Global p-value | 1  1  2 | 1.15  0.741  1.03  0.482  0.920 | |
| **Plant 1** | | | | | | | |
| **Unexposed Baseline** | **Obs** | **RR** | **95% CI** | **Low Exposed Baseline** | **Obs** | **RR** | **95% CI** |
| NCI Cats. Wald^k^  **Unexposed (Baseline)**  Exp Cat 1  Exp Cat 2  Exp Cat 3 | 0  4  0  1 | 1.00  9.15E+7  0.78  8.03E+8 | NA NA NA | NCI Cats. Wald  Unexposed  **Exp Cat 1 (Baseline)**  Exp Cat 2  Exp Cat 3 | 0  4  0  1 | 0  1.00  0  8.78 | NA  NA  (0.55,140.68) |
| Slope estimate (Score1)  Trend p-value (Score1)  Slope estimate (Score4)  Trend p-value (Score4)  Global p-value | 1  1  3 | 2.44  0.176  1.09  0.200  0.502 | | Slope estimate (Score1)  Trend p-value (Score1)  Slope estimate (Score4)  Trend p-value (Score4)  Global p-value | 1  1  2 | 2.08  0.313  1.08  0.231  0.308 | |
| UPitt Cats. Wald  **Unexposed (Baseline)**  Exp Cat 1  Exp Cat 2  Exp Cat 3 | 0  3  2  1 | 1.00  7.79E+6  7.61E+6  9.28E+6 | NA NA NA | UPitt Cats. Wald  Unexposed  **Exp Cat 1 (Baseline)**  Exp Cat 2  Exp Cat 3 | 0  3  2  1 | 0  1.00  0.98  1.19 | NA  (0.16,5.90) (0.12,12.29) |
| Slope estimate (Score1)  Trend p-value (Score1)  Slope estimate (Score4)  Trend p-value (Score4)  Global p-value | 1  1  3 | 1.33  0.565  1.09  0.176  0.999 | | Slope estimate (Score1)  Trend p-value (Score1)  Slope estimate (Score4)  Trend p-value (Score4)  Global p-value | 1  1  2 | 1.06  0.913  1.08  0.204  0.987 | |
| **Plants 2-10** | | | | | | | |
| **Unexposed Baseline** | **Obs** | **RR** | **95% CI** | **Low Exposed Baseline** | **Obs** | **RR** | **95% CI** |
| NCI Cats.  **Unexposed (Baseline)**  Exp Cat 1  Exp Cat 2  Exp Cat 3 | 2  1  1  1 | 1.00  0.11  0.35  0.41 | (0.01,1.53)  (0.03,4.58)  (0.03,7.52) | NCI Cats.  Unexposed  **Exp Cat 1 (Baseline)**  Exp Cat 2  Exp Cat 3 | 2  1  1  1 | 8.80  1.00  3.07  3.65 | (0.65,118.45)  (0.19,50.42)  (0.19,68.42) |
| Slope estimate (Score1)  Trend p-value (Score1)  Slope estimate (Score4)  Trend p-value (Score4)  Global p-value | 1  1  3 | 0.86  0.797  1.02  0.773  0.381 | | Slope estimate (Score1)  Trend p-value (Score1)  Slope estimate (Score4)  Trend p-value (Score4)  Global p-value | 1  1  2 | 1.79  0.400  1.04  0.530  0.586 | |
| UPitt Cats.  **Unexposed (Baseline)**  Exp Cat 1  Exp Cat 2  Exp Cat 3 | 2  0  1  2 | 1.00  0  0.20  0.41 | NA (0.02,2.62)  (0.04,4.00) | UPitt Cats.  Unexposed  **Exp Cat 1 (Baseline)**  Exp Cat 2  Exp Cat 3 | 2  0  1  2 | 8.40E+7  1.00  1.69E+7  3.44E+7 | NA  NA NA |
| Slope estimate (Score1)  Trend p-value (Score1)  Slope estimate (Score4)  Trend p-value (Score4)  Global p-value | 1  1  3 | 0.89  0.800  1.02  0.773  0.203 | | Slope estimate (Score1)  Trend p-value (Score1)  Slope estimate (Score4)  Trend p-value (Score4)  Global p-value | 1  1  2 | 3.52  0.153  1.04  0.530  0.282 | |

1. NCI categories based on 60^th^ and 80^th^ percentiles of FA exposure among cancer deaths who were exposed. Includes on 10/11 deaths.
2. UPitt categories based on approximate tertiles of FA exposure among NPC deaths who were exposed. Includes 11 deaths.
3. All exposures lagged 15 years as in NCI study
4. **Score1:** Assign 1,2,3,4 to the non-exposure, low, median and high exposure groups and treat the exposure as continuous in the model.
5. **Score2:** Pseudo-continuous PEAK score defined as the arithmetic mean of the peak interval, including a reasonable assumption about the score for the last open-ended interval (PEAK score : unexposed=0, >0-1.9=0.95, 2.0-3.9=3.0, 4.0+=6.0)
6. **Score4:** Real continuous exposure metrics (AIE, CUM & DUR)
7. **NCI exposure category cut points:** Highest peak (>0-1.9, 2.0-3.9, 4.0+ ppm); AIE (>0-<0.5, 0.5-<1.0, 1.0+ ppm); CUM(>0-<1.5, 1.5-<5.5, 5.5+ ppm-years); DUR (>0-<5.0, 5.0-<15.0, 15.0+ years), **UPitt exposure category cut points:** Highest peak same as NCI; AIE (>0-< 1.046, 1.046-<1.178, 1.178+ ppm); CUM (>0-<0.734, 0.734-<10.151, 10.151+ ppm-years); DUR (>0-<0.617, 0.617-<6.264, 6.264+ years)
8. All trend test and global test for exposure are among unexposed and exposed workers
9. All trend test and global test for exposure are among exposed workers
10. **N.C.:** Model did not coverage
11. **Wald:** The restricted model did not converge. Wald type test are used.

*p < 0.05

**p<0.01
