# Supplementary material for: An updated re-analysis of the mortality risk from nasopharyngeal cancer in the National Cancer Institute formaldehyde worker cohort study
Source: J Occup Med Toxicol. 2016 Mar 2;11:8. doi: 10.1186/s12995-016-0097-6 (PMC4774098; doi:10.1186/s12995-016-0097-6)
Supplement: Additional file 3: Table S3. — a Observed deaths and interval rate-based RRs or β coefficients by highest peak FA exposure. b Observed deaths and interval rate-based RRs or β coefficients by average intensity of FA exposure (ppm). c Observed deaths and interval rate-based RRs or β coefficients by cumulative FA exposure (ppm-years). d Observed deaths and interval rate-based RRs or β coefficients by duration of FA exposure (years). (DOCX 77 kb) [file 12995_2016_97_MOESM3_ESM.docx]

**Table S3a**

**Observed deaths and interval rate-based RRs^a^ or β coefficients^a^ by highest peak FA exposure^b,c,d^**

| **Model #** | **Model** | **Observed deaths** | **RR** | **95%CI for RR** | **Trend *p*-value** |
| --- | --- | --- | --- | --- | --- |
| 1 | *NCI categories (All plants)* |  |  |  |  |
|  | Unexposed | 2 | 1 |  |  |
|  | >0-1.9 | 1 | .234 | .004-5.07 | *p*^g^ = .073 *(Score 1^m^)* |
|  | 2.0-3.9 | 0 | .264^i^ | 0-2.3 | *p*^h^ = .004** *(Score 1)* |
|  | 4.0+ | 8 | 1.8 | .28-20.82 |  |
| 2 | *NCI categories^f^ (All plants)* |  |  |  |  |
|  | Unexposed | 2 | 1 |  |  |
|  | >0-1.9 | 1 | .307 | .004-7.17 | *p*^g^ = .263 *(Score 1)* |
|  | 2.0-3.9 | 0 | .224^i^ | 0-1.98 | *p*^h^ =.042* *(Score 1)* |
|  | 4.0+ | 8 | 1.392 | .19-17.43 |  |
|  |  |  |  |  |  |
|  | **Model** | **Observed deaths** | **β (RR)** | **95%CI for β**  **(Wald *p*-value)** | **Global *p*-value** |
| 3 | *Pseudo-continuous* PEAK *(Score 2^n^)(All plants)* | 11 | .3149 (1.37) | .03-.63 (.027*) | *P*^j^ = .083 |
| 4 | *Pseudo-continuous* PEAK *(Score 2)(Plants 2-10)* | 5 | .0072 (1.01) | -.45-.4 (1.000) | *P*^j^ = .666 |
| 5 | *Pseudo-continuous* PEAK *(Score 2)(Plants 1)* | 6 | .6381^i^ (1.89) | .13-∞ (.028*) | *P*^j^ = .054 |
| 6 | Plant group |  |  |  |  |
|  | Plants 2-10 (baseline) | 5 |  |  |  |
|  | Plant 1 | 6 | 1.5163 (4.56) | .13-2.95 (.031*) | *P*^k =^ .015* |
| 7 | *Pseudo-continuous* PEAK *(Score 2)(All plants)* |  | .2555 (1.29) | -.05-.59 (.102) | *P*^j^ = .267 |
|  | Plant group | 11 |  |  |  |
|  | Plants 2-10 (baseline) |  |  |  |  |
|  | Plant 1 |  | 1.1586 (3.19) | -.28-2.7 (.128) | *P*^k =^ .084 |
| 8 | *Pseudo-continuous* PEAK *(Score 2)(Plants 2-10)* |  | .0936 (1.1) | -.35-.48 (.697) | *P*^j^ = .909 |
|  | *Pseudo-continuous* PEAK *(Score 2)*plant group* | 11 | .3346 (1.4) | .05-.71 (.020*) | *P*^l^ = .03* |
| 9 | *Pseudo-continuous* PEAK *(Score 2)(Plants 2-10)* |  | .0223 (1.02) | -.43-.41 (.961) | *P*^j^ = .685 |
|  | Plant group | 11 |  |  |  |
|  | Plants 2-10 (baseline) |  |  |  |  |
|  | Plant 1 |  | -.7181^i^ (.49) | -∞-1.64 (.619) | *P*^k =^ .419 |
|  | *Continuous* PEAK**plant group* |  | .6121^i^ (1.84) | .03-∞ (.085) | *P*^l^ = .093 |

**Table S3b**

**Observed deaths and interval rate-based RRs^a^ or β coefficients^a^ by average intensity of FA exposure^b,c,d^ (ppm)**

| **Model #** | **Model** | **Observed deaths** | **RR** | **95%CI** | **Trend *p*-value** |
| --- | --- | --- | --- | --- | --- |
| 1 | *Upitt categories (all plants)* |  |  |  |  |
|  | Unexposed | 2 | 1 |  |  |
|  | >0-1.046 | 4 | .41 | .05-5.33 | *p*^g^ =.209 *(Score 1^m^)* |
|  | 1.047-1.117 | 2 | 1.74 | .11-26.67 | *p*^h^ =.056 *(Score 1)* |
|  | 1.118+ | 3 | 1.42 | .14-19.51 |  |
| 2 | *Upitt categories^f^ (All plants)* |  |  |  |  |
|  | Unexposed | 2 | 1 |  |  |
|  | >0-1.046 | 4 | .45 | .05-6.3 | *p*^g^ =.61 *(Score 1)* |
|  | 1.047-1.117 | 2 | .89 | .05-15.8 | *p*^h^ =.515 *(Score 1)* |
|  | 1.118+ | 3 | 1.01 | .09-15.14 |  |
|  |  |  |  |  |  |
|  | **Model** | **Observed deaths** | **β(RR)** | **95%CI**  **(Wald *p*-value)** | **Global *p*-value** |
| 3 | *Pseudo-continuous AIE (Score 3^o^)(All plants)* | 11 | .7745 (2.17) | -.21-1.74 (.115) | *P*^j^ =.101 |
| 4 | *Pseudo-continuous AIE (Score 3)(Plants 2-10)* | 5 | .2601 (1.30) | -2.24-1.81 (.828) | *P*^j^ =.193 |
| 5 | *Pseudo-continuous AIE (Score 3)(Plants 1)* | 6 | .3846 (1.47) | -1.02-1.92 (.651) | *P*^j^ = 1.000 |
| 6 | Plant group |  |  |  |  |
|  | Plants 2-10 (baseline) | 5 |  |  |  |
|  | Plant 1 | 6 | 1.5163 (4.56) | .13-2.95 (.030*) | *P*^k =^.015* |
| 7 | *Pseudo-continuous AIE (Score 3)(all plants)* |  | .3856 (1.47) | -.71-1.45 (.477) | *P*^j^ =.654 |
|  | Plant group | 11 |  |  |  |
|  | Plants 2-10 (baseline) |  |  |  |  |
|  | Plant 1 |  | 1.2478 (3.48) | -.33-2.84 (.134) | *P*^k =^.074 |
| 8 | *Pseudo-continuous AIE (Score 3)(Plants 2-10)* |  | .2921 (1.34) | -2.18-1.82 (.807) | *P*^j^ =.307 |
|  | *Pseudo-continuous AIE (Score 3)*plant group* | 11 | 1.0743 (2.93) | -.35-3.29 (.149) | *P*^l^ =.063 |
| 9 | *Pseudo-continuous AIE (Score 3)(Plants 2-10)* |  | .2404 (1.27) | -2.24-1.78 (.838) | *P*^j^ =.294 |
|  | Plant group | 11 |  |  |  |
|  | Plants 2-10 (baseline) |  |  |  |  |
|  | Plant 1 |  | .6318^i^ (1.88) | -Inf-2.78 (1.000) | *P*^k =^ 1.000 |
|  | *Pseudo-continuous AIE (Score 3)*plant group* |  | .0828 (1.09) | -1.86-2.95 (.92) | *P*^l^ =.484 |

**Table S3c**

**Observed deaths and interval rate-based RRs^a^ or β coefficients^a^ by cumulative FA exposure^b,c,d^ (ppm-years)**

| **Model #** | **Model** | **Observed deaths** | **RR** | **95%CI** | **Trend *p*-value** |
| --- | --- | --- | --- | --- | --- |
| 1 | *Upitt categories (all plants)* |  |  |  |  |
|  | Unexposed | 2 | 1.00 |  |  |
|  | >0-.734 | 4 | .65 | .08-8.35 | *p*^g^ =.437 *(Score 1^m^)* |
|  | .735-10.150 | 2 | .49 | .03-7.64 | *p*^h^ =.126 *(Score 1)* |
|  | 10.151+ | 3 | 2.84 | .25-43.38 |  |
| 2 | *Upitt categories^f^ (All plants)* |  |  |  |  |
|  | Unexposed | 2 | 1.00 |  |  |
|  | >0-.734 | 4 | .60 | .07-8.29 | *p*^g^ =.424 *(Score 1)* |
|  | .735-10.150 | 2 | .42 | .03-6.98 | *p*^h^ =.123 *(Score 1)* |
|  | 10.151+ | 3 | 2.99 | .23-49.56 |  |
|  |  |  |  |  |  |
|  | **Model** | **Observed deaths** | **β(RR)** | **95%CI**  **(Wald *p*-value)** | **Global *p*-value** |
| 3 | *Pseudo-continuous CUM (Score 3^o^)(All plants)* | 11 | .0940 (1.10) | -.01-.18 (.083) | *P*^j^ =.119 |
| 4 | *Pseudo-continuous CUM (Score 3)(Plants 2-10)* | 5 | .1183 (1.13) | -.04-.22 (.142) | *P*^j^ =.023* |
| 5 | *Pseudo-continuous CUM (Score 3)(Plants 1)* | 6 | .0951 (1.10) | -.1-.24 (.271) | *P*^j^ =.422 |
| 6 | Plant group |  |  |  |  |
|  | Plants 2-10 (baseline) | 5 |  |  |  |
|  | Plant 1 | 6 | 1.5163 (4.56) | .13-2.95 (.030*) | *P*^k =^.015* |
| 7 | *Pseudo-continuous CUM (Score 3)(all plants)* |  | .1030 (1.11) | -.01-.19 (.063) | *P*^j^ =.077 |
|  | Plant group | 11 |  |  |  |
|  | Plants 2-10 (baseline) |  |  |  |  |
|  | Plant 1 |  | 1.6060 (4.98) | .23-3.04 (.021*) | *P*^k =^.011* |
| 8 | *Pseudo-continuous CUM (Score 3)(Plants 2-10)* |  | .1276 (1.14) | -.03-.23 (.102) | *P*^j^ =.026* |
|  | *Continuous CUM*plant group* | 11 | .0818 (1.09) | -.03-.24 (.129) | *P*^l =^.004** |
| 9 | *Pseudo-continuous CUM (Score 3)(Plants 2-10)* |  | .1249 (1.13) | -.03-.23 (.109) | *P*^j^ =.017* |
|  | Plant group | 11 |  |  |  |
|  | Plants 2-10 (baseline) |  |  |  |  |
|  | Plant 1 |  | .5900^i^ (1.80) | -Inf-2.73 (1.000) | *P*^k =^ 1.000 |
|  | *Pseudo-continuous CUM (Score 3)*plant group* |  | -.0363 (.96) | -.18-.17 (1.000) | *P*^l =^.397 |

**Table S3d**

**Observed deaths and interval rate-based RRs^a^ or β coefficients^a^ by duration of FA exposure^b,c,d^ (years)**

| **Model #** | **Model** | **Observed deaths** | **RR** | **95%CI** | **Trend *p*-value** |
| --- | --- | --- | --- | --- | --- |
| 1 | *Upitt categories (All plants)* |  |  |  |  |
|  | Unexposed | 2 | 1.00 |  |  |
|  | >0-.616 | 3 | .69 | .07-9.65 | *p*^g^ = 1.000 *(Score 1^m^)* |
|  | .617-6.263 | 3 | .69 | .07-9.24 | *p*^h^ =.832 *(Score 1)* |
|  | 6.264+ | 3 | .88 | .08-13.32 |  |
| 2 | *Upitt categories^f^ (All plants)* |  |  |  |  |
|  | Unexposed | 2 | 1.00 |  |  |
|  | >0-.616 | 3 | .54 | .05-8.11 | *p*^g^ =.866 *(Score 1)* |
|  | .617-6.263 | 3 | .63 | .06-9.1 | *p*^h^ =.507 *(Score 1)* |
|  | 6.264+ | 3 | .99 | .08-16.18 |  |
|  |  |  |  |  |  |
|  | **Model** | **Observed deaths** | **β(RR)** | **95%CI (Wald *p*-value)** | **Global *p*-value** |
| 3 | *Pseudo-continuous DUR (Score 3^o^)(All plants)* | 11 | .0144 (1.01) | -.12-.13 (.838) | *P*^j^ =.974 |
| 4 | *Pseudo-continuous DUR (Score 3)(Plants 2-10)* | 5 | .0422 (1.04) | -.15-.19 (.759) | *P*^j^ =.299 |
| 5 | *Pseudo-continuous DUR (Score 3)(Plants 1)* | 6 | .0244 (1.03) | -.20-.20 (.715) | *P*^j^ = 1.000 |
| 6 | Plant group |  |  |  |  |
|  | Plants 2-10 (baseline) | 5 |  |  |  |
|  | Plant 1 | 6 | 1.5163 (4.56) | .13-2.95 (.030*) | *P*^k =^.015* |
| 7 | *Pseudo-continuous DUR (Score 3)(all plants)* |  | .0366 (1.04) | -.10-.15 (.577) | *P*^j^ =.797 |
|  | Plant group | 11 |  |  |  |
|  | Plants 2-10 (baseline) |  |  |  |  |
|  | Plant 1 |  | 1.5912 (4.91) | .19-3.04 (.025*) | *P*^k =^.012* |
| 8 | *Pseudo-continuous DUR (Score 3)(Plants 2-10)* |  | .0583 (1.06) | -.13-.2 (.595) | *P*^j^ =.357 |
|  | *Pseudo-continuous DUR (Score 3)*plant group* | 11 | .1212 (1.13) | -.03-.32 (.094) | *P*^l^ =.005** |
| 9 | *Pseudo-continuous DUR (Score 3)(Plants 2-10)* |  | .0541 (1.06) | -.14-.20 (.634) | *P*^j^ =.232 |
|  | Plant group | 11 |  |  |  |
|  | Plants 2-10 (baseline) |  |  |  |  |
|  | Plant 1 |  | .6018 (1.83) | -Inf-2.75 (1.000) | *P*^k =^.1.000 |
|  | *Pseudo-continuous DUR (Score 3)*plant group* |  | -.0239 (.98) | -.25-.23 (.922) | *P*^l^ =.424 |

1. All models adjusted for age, time, sex, race and pay type.
2. NCI categories taken from Hauptmann et al. (2004).
3. UPitt categories based on approximate tertiles of FA exposure among NPC deaths who were exposed. Includes 11 deaths.
4. All exposures lagged 15 years.
5. Plant grouped as Plant 1 (code=1) vs. Plant 2-10 (code=0)
6. Model adjusted for plant group
7. Likelihood ratio test(one degree of freedom) for continuous score FA exposure among unexposed and exposed workers
8. Likelihood ratio test(one degree of freedom) for continuous score FA exposure among exposed workers
9. Median unbiased estimate.
10. Likelihood ratio test for Pseudo-continuous metric (Score 2)(tests the addition of the variable to a base model with adjustment factors).
11. Likelihood ratio test for plant group (tests the addition of the variable to a base model with adjustment factors)
12. Likelihood ratio test for interaction term (tests the addition of the variable to a base model with adjustment factors)
13. **Score 1:** Assign 1,2,3,4 to the non-exposure, low, median and high exposure groups and treat the exposure as continuous in the model.
14. **Score2:** Pseudo-continuous PEAK score defined as the arithmetic mean of the peak interval, including a reasonable assumption about the score for the last open-ended interval (unexposed=0, >0-1.9=0.95, 2.0-3.9=3.0, 4.0+=6.0)
15. **Score3**: Pseudo-continuous AIE, CUM and DUR score defined as the median value of each of the Upitt categories taken from Marsh et al. (2004) AIE (No exposure=0, >0-< 1.046=.21, 1.046-<1.178=1.10, 1.178+=1.55); CUM (No exposure=0, >0-<0.734=0.14, 0.734-<10.151=2.36, 10.151+=16.34); DUR (No exposure=0, >0-<0.617=0.25, 0.617- <6.264=2, 6.264+=13.01).

*p < 0.05

**p<0.01
