# Supplementary material for: An updated re-analysis of the mortality risk from nasopharyngeal cancer in the National Cancer Institute formaldehyde worker cohort study
Source: J Occup Med Toxicol. 2016 Mar 2;11:8. doi: 10.1186/s12995-016-0097-6 (PMC4774098; doi:10.1186/s12995-016-0097-6)
Supplement: Additional file 4: Table S4. — a Observed deaths and interval RRs or β coefficients by peak FA exposure, asymptotic estimation. b Observed deaths and interval RRs or β coefficients by average FA intensity exposure, asymptotic estimation. c Observed deaths and RRs or β coefficients by cumulative FA exposure, asymptotic estimation. d Observed deaths and RRs or β coefficients by duration of FA exposure, asymptotic estimation. (DOCX 66 kb) [file 12995_2016_97_MOESM4_ESM.docx]

**Supplementary Table S4-a**

**Observed deaths and interval RRs^a^ or β coefficients^a^ by peak FA exposure^b,c,d^, asymptotic estimation**

| **Model #** | **Model** | **Observed deaths** | **RR** | **95%CI** | **Trend *p*-value** |
| --- | --- | --- | --- | --- | --- |
| 1 | *NCI categories (all plants)* |  |  |  |  |
|  | Unexposed | 2 | 1 |  |  |
|  | >0-1.9 | 1 | 0.220 | (0.02-2.72) | *p*^g^ = 0.060 (*Score 1*) |
|  | 2.0-3.9 | 0 | 3.38E-08 | (0-Inf) | *p*^h^ = 0.003** (*Score 1*) |
|  | 4.0+ | 8 | 1.800 | (0.32-10.04) |  |
| 2 | *NCI categories^f^ (All plants)* |  |  |  |  |
|  | Unexposed | 2 | 1 |  |  |
|  | >0-1.9 | 1 | 0.290 | (0.02-3.89) | *p*^g^ = 0.201 (*Score 1*) |
|  | 2.0-3.9 | 0 | 2.58E-08 | (0-Inf) | *p*^h^ = 0.022* (*Score 1*) |
|  | 4.0+ | 8 | 1.390 | (0.23-8.41) |  |
|  |  |  |  |  |  |
|  | **Model** | **Observed deaths** | **β(RR)** | **95%CI (Wald *p*-value)** | **Gkobak *p*-vakue** |
| 3 | *Continuous peak (Score 2 ^i^)*  *(All plants)* | 11 | 0.315 (1.37) | 1.04-1.82 (0.03*) | *p*^i^ = 0.020* |
| 4 | *Continuous peak (Score 2)*  *(Plants 2-10)* | 5 | 0.007 (1.01) | .68-1.49 (0.97) | *p*^i^ = 0.964 |
| 5 | *Continuous peak (Score 2)*  *(Plants 1)* | 6 | 5.721 (305.20) | 0-Inf (0.99) | *p*^i^ = 0.003** |
| 6 | Plant group |  |  |  |  |
|  | Plants 2-10 (baseline) | 5 |  |  |  |
|  | Plant 1 | 6 | 1.517 (4.56) | 1.37-15.14 (0.01*) | *p*^j^ = 0.015** |
| 7 | *Continuous peak (Score 2)*  *(all plants)* |  | 0.256 (1.29) | .96-1.74 (0.09) | *p*^i^ = 0.080 |
|  | Plant group | 11 |  |  |  |
|  | Plants 2-10 (baseline) |  |  |  |  |
|  | Plant 1 |  | 1.194 (3.30) | 0.96-11.35 (0.06) | *p*^j^ = 0.059 |
| 8 | *Continuous peak (Score 2)*  *(Plants 2-10)* |  | 0.103 (1.11) | 0.76-1.62 (0.59) | *p*^i^ = 0.601 |
|  | *Continuous peak (Score 2)*plant group* | 11 | 0.336 (1.40) | 1.05-1.86 (0.02*) | *p*^k^ = 0.011* |
| 9 | *Continuous peak (Plants 2-10)* |  | 0.022 (1.02) | 0.69-1.51 (0.91) | *p*^i^ = 0.913 |
|  | Plant group | 11 |  |  |  |
|  | Plants 2-10 (baseline) |  |  |  |  |
|  | Plant 1 |  | -31.295  (2.56E-14) | 0-Inf (0.99) | *p*^j^ = 0.087 |
|  | *Continuous peak (Score 2) *plant group* |  | 5.584 (266.11) | 0-Inf (0.99) | *p*^k^ = 0.015* |

**Supplementary Table S4-b**

**Observed deaths and interval RRs^a^ or β coefficients^a^ by average FA intensity exposure^b,c,d^, asymptotic estimation**

| **Model #** | **Model** | **Observed deaths** | **RR** | **95%CI** | **Trend *p*-value** |
| --- | --- | --- | --- | --- | --- |
| 1 | *Upitt categories (all plants)* |  |  |  |  |
|  | Unexposed | 2 | 1 |  |  |
|  | >0-1.046 | 4 | 0.402 | 0.06-2.59 | *p*^g^ = 0.225 (*Score 1*) |
|  | 1.047-1.117 | 2 | 1.674 | 0.21-13.17 | *p*^h^ = 0.078 (*Score 1*) |
|  | 1.118+ | 3 | 1.389 | 0.20-9.51 |  |
| 2 | *Upitt categories^f^ (All plants)* |  |  |  |  |
|  | Unexposed | 2 | 1 |  |  |
|  | >0-1.046 | 4 | 0.446 | 0.07-3.03 | *p*^g^ = 0.618 (*Score 1*) |
|  | 1.047-1.117 | 2 | 0.874 | 0.10-7.95 | *p*^h^ = 0.433 (*Score 1*) |
|  | 1.118+ | 3 | 0.993 | 0.13-7.38 |  |
|  |  |  |  |  |  |
|  | **Model** | **Observed deaths** | **β(RR)** | **95%CI**  **(Wald *p*-value)** | **Global *p*-value** |
| 3 | *Continuous AIE (Score 4 ^i^)*  *(All plants)* | 11 | 0.524 (1.69) | 0.99-2.88 (0.05) | *P*^j^ = 0.087 |
| 4 | *Continuous AIE (Score 4) (Plants 2-10)* | 5 | 0.408 (1.50) | 0.68-3.32 (0.31) | *P*^j^ = 0.364 |
| 5 | *Continuous AIE (Score 4) (Plants 1)* | 6 | 2.287 (9.84) | 0.62-156.61 (0.11) | *P*^j^ = 0.071 |
| 6 | Plant group |  |  |  |  |
|  | Plants 2-10 (baseline) | 5 |  |  |  |
|  | Plant 1 | 6 | 1.517 (4.56) | 1.37-15.14 (0.01*) | *P*^k^ = 0.015* |
| 7 | *Continuous AIE (Score 4)*  *(all plants)* |  | 0.563 (1.76) | 0.92-3.37 (0.09) | *P*^j^ = 0.132 |
|  | Plant group | 11 |  |  |  |
|  | Plants 2-10 (baseline) |  |  |  |  |
|  | Plant 1 |  | 1.424 (4.15) | 1.25-13.79 (0.02*) | *P*^k^ = 0.022* |
| 8 | *Continuous AIE (Score 4) (Plants 2-10)* |  | 0.430 (1.54) | 1.52-3.28 (0.27) | *P*^j^ = 0.325 |
|  | *Continuous AIE (Score 4)*plant group* | 11 | 1.406 (4.08) | 0.21-10.98 (0.01**) | *P*^l^ = 0.007** |
| 9 | *Continuous AIE (Score 4) (Plants 2-10)* |  | 0.397 ( 1.49) | 0.68-3.24 (0.32) | *P*^j^ = 0.372 |
|  | Plant group | 11 |  |  |  |
|  | Plants 2-10 (baseline) |  |  |  |  |
|  | Plant 1 |  | -0.686 (0.50) | 0.02-16.31 (0.70) | *P*^k^ = 0.691 |
|  | *Continuous AIE (Score 4)*plant group* |  | 1.918 (6.81) | 0.43-107.18 (0.17) | *P*^l^ = 0.142 |

**Supplementary Table S4-c**

**Observed deaths and RRs^a^ or β coefficients^a^ by cumulative FA exposure^b,c,d^, asymptotic estimation**

| **Model #** | **Model** | **Observed deaths** | **RR** | **95%CI** | **Trend *p*-value** |
| --- | --- | --- | --- | --- | --- |
| 1 | *Upitt categories (all plants)* |  |  |  |  |
|  | Unexposed | 2 | 1 |  |  |
|  | >0-.734 | 4 | 0.635 | 0.10-4.02 | *p*^g^ = 0.382 (*Score 1*) |
|  | .735-10.150 | 2 | 0.464 | 0.06-3.75 | *p*^h^ = 0.137 (*Score 1*) |
|  | 10.151+ | 3 | 2.800 | 0.37-21.26 |  |
| 2 | *Upitt categories^f^ (All plants)* |  |  |  |  |
|  | Unexposed | 2 | 1 |  |  |
|  | >0-.734 | 4 | 0.588 | 0.09-3.95 | *p*^g^ = 0.389 (*Score 1*) |
|  | .735-10.150 | 2 | 0.396 | 0.05-3.39 | *p*^h^ = 0.135 (*Score 1*) |
|  | 10.151+ | 3 | 2.941 | 0.36-24.27 |  |
|  |  |  |  |  |  |
|  | **Model** | **Observed deaths** | **β(RR)** | **95%CI**  **(Wald *p*-value)** | **Global *p*-value** |
| 3 | *Continuous CUM (Score 4 ^i^)*  *(All plants)* | 11 | 0.035 (1.04) | 1.00-1.07 (0.04*) | *P*^j^ = 0.091 |
| 4 | *Continuous CUM (Score 4)*  *(Plants 2-10)* | 5 | 0.037 (1.04) | 1.00-1.08 (0.09) | *P*^j^ = 0.151 |
| 5 | *Continuous CUM (Score 4)*  *(Plants 1)* | 6 | 0.147 (1.16) | 1.03-1.31 (0.02*) | *P*^j^ = 0.040* |
| 6 | Plant group |  |  |  |  |
|  | Plants 2-10 (baseline) | 5 |  |  |  |
|  | Plant 1 | 6 | 1.5170 (4.56) | 1.37-15.14 (.01*) | *P*^k^ = 0.015* |
| 7 | *Continuous CUM (Score 4)*  *(all plants)* |  | 0.045 (1.05) | 1.01-1.08 (0.01*) | *P*^j^ = 0.043* |
|  | Plant group | 11 |  |  |  |
|  | Plants 2-10 (baseline) |  |  |  |  |
|  | Plant 1 |  | 1.735 (5.67) | 1.60-20.14 (0.01**) | *P*^k^ = 0.008** |
| 8 | *Continuous CUM (Score 4)*  *(Plants 2-10)* |  | 0.033 (1.03) | 0.99-1.07 (0.11) | *P*^j^ = 0.191 |
|  | *Continuous CUM (Score 4)*plant group* | 11 | 0.144 (1.16) | 1.06-1.27 (0.002**) | *P*^l^ = 0.015* |
| 9 | *Continuous CUM (Score 4)*  *(Plants 2-10)* |  | 0.040 (1.04) | 1.00-1.08 (0.04*) | *P*^j^ = 0.112 |
|  | Plant group | 11 |  |  |  |
|  | Plants 2-10 (baseline) |  |  |  |  |
|  | Plant 1 |  | 1.296 (3.65) | 0.86-15.44 (0.08) | *P*^k^ = 0.086 |
|  | *Continuous CUM (Score 4)*plant group* |  | 0.086 (1.09) | 0.97-1.22 (0.15) | *P*^l^ = 0.186 |

**Supplementary Table S4-d**

**Observed deaths and RRs^a^ or β coefficients^a^ by duration of FA exposure^b,c,d^, asymptotic estimation**

| **Model #** | **Model** | **Observed deaths** | **RR** | **95%CI** | **Trend *p*-value** |
| --- | --- | --- | --- | --- | --- |
| 1 | *Upitt categories (All plants)* |  |  |  |  |
|  | Unexposed | 2 | 1 |  |  |
|  | >0-.616 | 3 | 0.676 | 0.10-4.69 | *p*^g^ = 1.000 (*Score 1*) |
|  | .617-6.263 | 3 | 0.669 | 0.10-4.46 | *p*^h^ = 0.740 (*Score 1*) |
|  | 6.264+ | 3 | 0.871 | 0.12-6.52 |  |
| 2 | *Upitt categories^f^ (All plants)* |  |  |  |  |
|  | Unexposed | 2 | 1 |  |  |
|  | >0-.616 | 3 | 0.527 | 0.07-3.91 | *p*^g^ = 0.815 (*Score 1*) |
|  | .617-6.263 | 3 | 0.609 | 0.09-4.35 | *p*^h^ = 0.411 (*Score 1*) |
|  | 6.264+ | 3 | 0.965 | 0.12-7.82 |  |
|  |  |  |  |  |  |
|  | **Model** | **Observed deaths** | **β(RR)** | **95%CI**  **(Wald *p*-value)** | **Global *p*-value** |
| 3 | *Continuous DUR (Score 4 ^i^)*  *(All plants)* | 11 | 0.026 (1.03) | 0.95-1.11 (0.52) | *P*^j^ = 0.531 |
| 4 | *Continuous DUR (Score 4)*  *(Plants 2-10)* | 5 | 0.016 (1.02) | 0.91-1.13 (0.77) | *P*^j^ = 0.773 |
| 5 | *Continuous DUR (Score 4)*  *(Plants 1)* | 6 | 0.083 (1.09) | 0.96-1.22 (0.18) | *P*^j^ = 0.217 |
| 6 | Plant group |  |  |  |  |
|  | Plants 2-10 (baseline) | 5 |  |  |  |
|  | Plant 1 | 6 | 1.5170 (4.56) | 1.37-15.14 (.01*) | *P*^k^ = 0.015* |
| 7 | *Continuous DUR (Score 4)*  *(all plants)* |  | 0.043 (1.04) | 0.97-1.13 (0.29) | *P*^j^ = 0.308 |
|  | Plant group | 11 |  |  |  |
|  | Plants 2-10 (baseline) |  |  |  |  |
|  | Plant 1 |  | 1.626 (5.09) | 1.50-17.24 (0.01**) | *P*^k^ = 0.011* |
| 8 | *Continuous DUR (Score 4)*  *(Plants 2-10)* |  | -0.006 (0.99) | 0.90-1.10 (0.91) | *P*^j^ = 0.913 |
|  | *Continuous DUR (Score 4) *plant group* | 11 | 0.105 (1.11) | 0.99-1.24 (0.07) | *P*^l^ = 0.088 |
| 9 | *Continuous DUR (Score 4)*  *(Plants 2-10)* |  | 0.030 (1.03) | 0.93-1.14 (0.58) | *P*^j^ = 0.592 |
|  | Plant group | 11 |  |  |  |
|  | Plants 2-10 (baseline) |  |  |  |  |
|  | Plant 1 |  | 1.455 (4.29) | 0.99-18.48 (0.05) | *P*^k^ = 0.052 |
|  | *Continuous DUR (Score 4)*plant group* |  | 0.028 (1.03) | 0.90-1.18 (0.17) | *P*^l^ = 0.686 |

1. All models adjusted for age, time, sex, race and pay type.
2. NCI categories taken from Hauptmann et al. (2004).
3. UPitt categories based on approximate tertiles of FA exposure among NPC deaths who were exposed. Includes 11 deaths.
4. All exposures lagged 15 years.
5. Plant grouped as Plant 1 (code=1) vs. Plant 2-10 (code=0)
6. Model adjusted for plant group
7. Likelihood ratio test(one degree of freedom) for continuous score FA exposure among unexposed and exposed workers
8. Likelihood ratio test(one degree of freedom) for continuous score FA exposure among exposed workers
9. **Score 1:** Assign 1,2,3,4 to the non-exposure, low, median and high exposure groups and treat the exposure as continuous in the model.

**Score 4:** Real continuous exposure metrics (AIE, CUM & DUR)

1. Likelihood ratio test for continuous peak (tests the addition of the variable to a base model with adjustment factors).
2. Likelihood ratio test for plant group (tests the addition of the variable to a base model with adjustment factors)
3. Likelihood ratio test for interaction term (tests the addition of the variable to a base model with adjustment factors)

*p < 0.05

**p<0.01
